# Dynamic Cultivation of *Akkermansia muciniphila* in an Improved Gastrointestinal Reactor: Enhanced Growth and Metabolomic Profiling

**DOI:** 10.3390/foods15091467

**Published:** 2026-04-22

**Authors:** Yuqin Wang, Kexin Yu, Tongyan Shen, Kunqing Huang, Mengdie Li, Yating Wang, Jiaqi Xi, Jintian Chen, Minjie Gao, Zhitao Li

**Affiliations:** 1 School of Grain Science and Technology, Jiangsu University of Science and Technology, Zhenjiang 212100, China; yqwang@just.edu.cn (Y.W.);; 2 State Key Laboratory of Food Science and Resources, Jiangnan University, Wuxi 214122, China

**Keywords:** probiotic, *Akkermansia muciniphila*, dynamic cultivation, gastrointestinal reactor, metabolomics, short-chain fatty acids

## Abstract

*Akkermansia muciniphila*, a next-generation probiotic in the human intestinal mucus layer, exhibits significant health-promoting properties. However, traditional static culture systems fail to replicate the dynamic peristaltic environment of the gastrointestinal tract, limiting understanding of its metabolic characteristics. This study employed an improved gastrointestinal bioreactor simulating intestinal peristalsis to investigate *A. muciniphila* growth dynamics and metabolomic profiles under dynamic conditions. Dynamic cultivation significantly enhanced bacterial growth. Biomass reached 1.32 ± 0.03 g/L in bovine heart infusion (BHI) medium and 2.03 ± 0.05 g/L in BHI supplemented with 2.5 g/L porcine mucin. These values represent increases of 45.05% and 123.08% relative to static BHI cultures, respectively. Dynamic conditions markedly elevated short-chain fatty acid production (acetic, propionic, isobutyric, isovaleric acids). Untargeted metabolomics identified 1463 metabolites with 1294 showing significant differential expression. Dynamic cultivation substantially altered amino acid biosynthesis, fatty acid, purine, and pyrimidine metabolism. These findings advance the understanding of *A. muciniphila* physiology and provide insights into its metabolic characteristics under simulated intestinal conditions.

## 1. Introduction

The human intestine harbors a diverse and highly complex community of microorganisms collectively called the gut microbiota. These microorganisms participate in material exchange and energy transfer with enterocytes while also playing a crucial role in modulating immune responses [1]. Epithelial cells form a barrier between the gut microbiota and host tissue, thereby modulating the host’s response to commensal gut bacteria [2].These cells serve multiple crucial functions: they establish protective mucosal barriers, produce an array of immunological mediators, and facilitate the transport of microbial antigens [3]. The physical and chemical components of the mucosal defense system establish a critical separation between the microbial communities and host immune structures, thereby preventing aberrant immune responses against beneficial gut microorganisms that could trigger inflammatory conditions in the intestinal tissue [4]. The gut microbiota tends to reside exclusively in the outer mucus layer, while the inner layer functions to maintain a physical between the gut microbiota and epithelial cells.

Among the diverse gut microbiota, *A. muciniphila* stands out as a particularly abundant and important species. As a member of the phylum *Verrucomicrobia*, *A. muciniphila* is the sole representative of this phylum found in gastrointestinal samples [5]. Remarkably, *A. muciniphila* accounts for approximately 1–4% of the total fecal microbiota in healthy individuals, making it one of the most abundant single species in the human intestinal tract [6,7]. This bacterium is present in approximately 90% of healthy humans and colonizes the intestinal tract early in life, reaching adult-like levels within the first year [8]. Its abundance typically increases from the neonatal period to adulthood, serving as an important indicator of gut microbiota vitality and biodiversity [9]. By the consuming mucus secreted by intestinal epithelial cells, it not only sustains its own population growth but also generates beneficial short-chain fatty acids (SCFAs), which play a crucial role in microbiota-gut-brain crosstalk [10]. Beyond SCFA production, *A. muciniphila* provides direct metabolic benefits to its host, functioning as an energy provider, synthesizing essential B and K vitamins, and participating in bile acid metabolism [11,12]. Notably, a higher *A. muciniphila* abundance is generally associated with a healthier gut environment and has been observed in centenarians, suggesting its potential role in longevity [13]. Conversely, a reduced *A. muciniphila* abundance has been consistently observed in individuals with metabolic disorders, inflammatory bowel disease, obesity, and type 2 diabetes, establishing this bacterium as a promising health biomarker [14,15].

The human gut microbiota is predominantly composed of obligate anaerobes that can be isolated and cultured using nutrient-rich or semi-complex media. However, *A. muciniphila* represents a distinctive obligate anaerobe that poses significant difficulties for isolation and laboratory cultivation [16]. This bacterium is highly sensitive to oxygen and temperature fluctuations, exhibiting a prolonged growth cycle and low viable cell counts in vitro. *A. muciniphila* grows relatively slowly in BHI medium, with its growth rate significantly lower than that observed in mucin-containing media [17]. Traditional *A. muciniphila* cultures are typically grown in liquid media under strictly anaerobic conditions in a static system. Li et al. [18] investigated the growth-promoting effect of Mulberry Galacto-Oligosaccharide on *A. muciniphila* using static culture methods. Wu et al. [19] studied high-density culture strategies for *A. muciniphila* using a shaking incubator. Given that the human intestinal tract undergoes constant peristaltic motion representing a dynamic physiological process, traditional static culture systems cannot effectively mimic the in vivo physiological state of *A. muciniphila*. Several dynamic in vitro gut simulation systems have been developed for studying gut microbiota, including the Simulator of the Human Intestinal Microbial Ecosystem (SHIME) [20] and the TNO Intestinal Model (TIM-2) [21]. These two systems are sophisticated multi-chamber models designed to simulate the entire gastrointestinal tract and complex microbial communities. Our gastrointestinal bionic reactor is a specialized, single-chamber bionic bioreactor that focuses specifically on the metabolic reprogramming of a single strain under mechanical peristaltic stress. It offers a more cost-effective, high-throughput, and controllable environment for the optimization and mechanistic studies of individual “next-generation probiotics” like *A. muciniphila*.

Metabolomics is a powerful tool for investigating the complex metabolic relationships between the gut microbiota and their hosts. Recent advancements in metabolomics have enabled the identification and quantification of diverse metabolites produced by the gut microbiota, providing insights into the metabolic activities of specific bacterial species and their potential roles in health and disease [22,23]. Unlike genomics and transcriptomics, which reveal genetic potential and gene expression, metabolomics directly captures the functional endpoint of cellular processes by measuring the complete set of small-molecule metabolites, thereby providing a real-time snapshot of the physiological state of an organism under specific conditions [24]. While our previous study established the feasibility of dynamic culture [25], the specific metabolic mechanisms remain unexplored. This study aims to systematically investigate the growth characteristics and metabolic profile changes of *A. muciniphila* under dynamic culture conditions using a gastrointestinal bionic reactor that simulates intestinal peristalsis. Combined with non-targeted metabolomics technology, this research seeks to reveal the mechanisms of the strain’s metabolic reprogramming. Compared to traditional static culture, dynamic culture more closely mimics the intestinal physiological environment, offering greater potential to accurately reflect the metabolic behavior of this bacterium within the host. Furthermore, by comparing the effects of porcine and human mucin on strain growth and metabolism, this study not only provides a basis for optimizing culture processes but also offers a new systems biology perspective for deepening our understanding of *A. muciniphila*’s metabolic adaptability and probiotic functional mechanisms. The innovation of this study lies in integrating dynamic bio-mimetic culture, mucin supplementation, and global metabolomics analysis.

## 2. Materials and Methods

### 2.1. Strain and Media

*A. muciniphila* AT56, isolated from Chinese human fecal samples in 2021 and previously described by Li et al. [25], is maintained in the in-house Culture Collection of Food Microbiology, Jiangnan University (Wuxi, China). Briefly, the *A. muciniphila AT56* strain was isolated from fresh fecal samples pooled from 20 healthy adult volunteers of Chinese origin (age range: 18–35 years; 10 males and 10 females). All donors had no history of gastrointestinal diseases, and had not received any antibiotic treatment within the three months preceding sample collection. Written informed consent was obtained from each participant prior to sample collection. The study was approved by the Human Ethics Committee of the Affiliated Hospital of Jiangnan University (approval No. S2022-003-10).

The experiment utilized three culture media: Brain Heart Infusion (BHI) medium, BHI supplemented with 2.5 g/L of porcine mucin (BPM), and BHI supplemented with 2.5 g/L of human mucin (BHM). The BHI medium contained the following: 10.0 g/L of tryptone (Sinopharm, Beijing, China), 2.5 g/L of Na_2_HPO_4_ (Sinopharm, Beijing, China), 17.5 g/L of bovine heart infusion (Hopebio, Qingdao, China), 5.0 g/L of NaCl (Sinopharm, Beijing, China), 2.0 g/L of glucose (Sinopharm, Beijing, China), and 1 L deionized water, adjusted to pH 6.5. Porcine mucin (Sigma-Aldrich, St. Louis, MO, USA) and human mucin (EnkiLife, Wuhan, China) were added as key carbon and nitrogen sources.

To ensure bacterial growth under anaerobic conditions, all media were prepared in an oxygen-free environment. The specific procedure involved boiling the prepared medium for 15 min under a continuous nitrogen gas flow to remove dissolved oxygen, followed by autoclaving (121 °C, 20 min) and cooling to 50 °C. To maintain anaerobic conditions, the medium was supplemented with the reducing agent L-cysteine hydrochloride (0.5 g/L; Sigma-Aldrich, St. Louis, MO, USA) and the redox indicator resazurin (1.0 mg/L; Sigma-Aldrich, St. Louis, MO, USA). This ensured that the redox state of the medium was suitable for the growth of anaerobic microorganisms.

### 2.2. Static and Dynamic Culture

**Figure 1:**
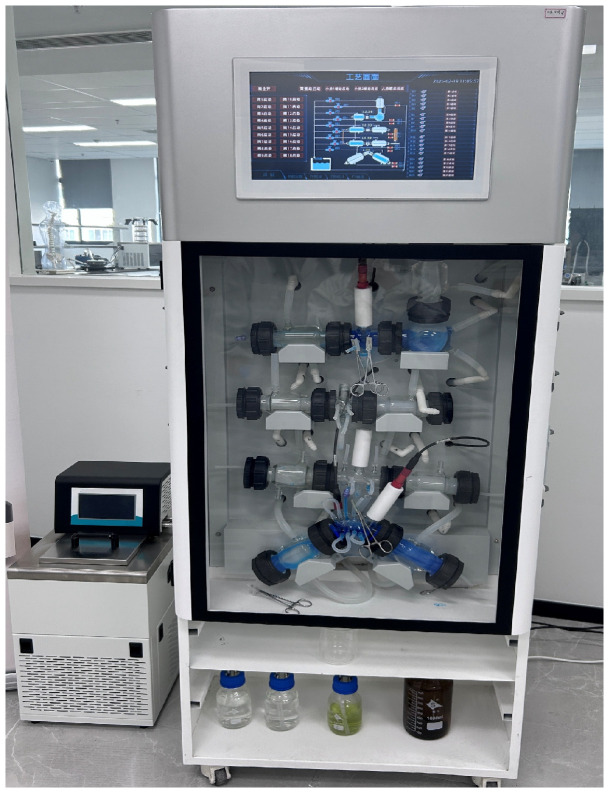
Picture of the gastrointestinal reactor.

This study employed a gastrointestinal reactor (Figure 1) for cultivation, with its core innovation lying in the integration of physiological peristalsis simulation. After assembling all components, plastic tubing was connected to each port and secured with hemostatic clamps, followed by submerging the entire apparatus in water. One clamp was released to introduce gas. Ventilation ceased when the colonic membrane began to inflate, and the clamp was reapplied. The absence of microbubbles indicated a properly sealed system. Subsequently, the reactor vessel, silicone membrane, connecting tubes, and all metal components were assembled and autoclaved at 121 °C for 30 min. After cooling to room temperature, the system was flushed with N_2_ to establish an anaerobic environment. For the dynamic culture group, initiate a customized peristaltic program to mimic human colonic motility. Building upon our previous design [25], this upgraded reactor integrates a 20 W servo-motor system to facilitate precise, bio-mimetic peristaltic contractions. The key parameters of the servo motor optimized from Reference [26], frequency of 0.1 Hz (6 cycles/minute), each cycle comprising a 4-s active contraction phase and a 6-s relaxation phase. The inner diameter of the colon-mimicking latex membrane was 30 mm with a wall thickness of 2 mm. *A. muciniphila AT56* was pre-cultured in the aforementioned anaerobic BHI medium at 37 °C until the late logarithmic phase (approximately 16–18 h). The pre-culture was inoculated into the reactor at 2% (*v*/*v*) once it reached an optical density (OD_600_) of approximately 1.5. The reactor utilized a 600 mL working volume with a 100 mL headspace of high-purity N_2_. Initial anaerobic conditions were established via a 30 min pre-flush (N_2_, 1.0 L/min). Samples (25 mL) were withdrawn via a sterile port every 8–12 h. After each sampling, 25 mL of fresh medium was replenished to maintain a constant working volume. The pH was continuously monitored and automatically maintained at 6.5 by the addition of 1 mol/L HCl (Aladdin, Shanghai, China) and 1 mol/L NaOH (Aladdin, Shanghai, China) solutions as needed. The static culture group was maintained under identical conditions without activating peristalsis. The culture was maintained at 37 °C throughout, with baseline parameters recorded online via an integrated pH sensor.

### 2.3. Cell Biomass Assay

Biomass concentration was determined gravimetrically as dry cell weight (DCW). Culture samples (20 mL) were collected at designated time points and centrifuged at 8000× *g* for 10 min at 4 °C. Cell pellets were washed twice with sterile phosphate buffered saline (PBS, pH 7.4, Aladdin, Shanghai, China) to remove residual medium components. The washed pellets were transferred to pre-weighed aluminum pans and dried at 105 °C for 24 h until a constant weight was achieved.

### 2.4. SCFAs Measurement

The determination of SCFAs (including acetic acid, propionic acid, butyric acid, isobutyric acid, isovaleric acid and valeric acid) was performed following ether extraction and subsequent analysis. The procedure was performed as follows: After centrifugation of the fermented samples at 8000 r/min for 5 min, 1.0 mL of the supernatant was collected. Then, 250 μL of HCl and 1.0 mL of anhydrous diethyl ether were added, followed by vortex mixing for 3 min and centrifugation to separate the organic phase. The organic phase was filtered through a 0.22 μm organic-compatible membrane into a 1.5 mL microcentrifuge tube, and anhydrous sodium sulfate was added to remove residual moisture. Subsequent detection was carried out using an Agilent 7820A GC system. Compound separation was conducted on an HP-INNOWAX capillary column (30 m × 0.25 mm i.d., 0.25 µm, 19091N-133). The GC temperature profile initiated at 60 °C for 1 min, then increased to 190 °C at a rate of 20 °C/min, maintaining this temperature for 7.5 min. Operating parameters included an injector temperature of 220 °C and flame ionization detector (FID) temperature of 250 °C. Five-microliter samples were introduced using a 20:1 split ratio. High-purity nitrogen (>99.999% purity) was used as the carrier gas with a constant column flow rate of 1.5 mL/min. Hydrogen and air flow rates were 40 mL/min and 400 mL/min, respectively. The concentrations of acetic acid, propionic acid, butyric acid, isobutyric acid, isovaleric acid, and valeric acid were determined based on regression equations derived from GC standards.

### 2.5. Metabolites Extraction

To comprehensively analyze metabolic reprogramming, we performed non-targeted metabolomics analysis based on UHPLC-MS/MS. A 100 μL aliquot of the whole culture medium (containing cells) was mixed with 400 μL of extraction solvent (methanol:water:formic acid = 80:19.9:0.1, *v*/*v*/*v*) pre-chilled to −40 °C. After vortexing on ice for 5 min, the mixture was centrifuged at 4 °C and 15,000× *g* for 10 min. The supernatant was collected and diluted with LC-MS grade water to a methanol concentration of 53%. The diluted sample was centrifuged again at 4 °C and 15,000× *g* for 10 min. The resulting supernatant was used for instrument analysis. To monitor system stability and reproducibility, prepare quality control (QC) samples were prepared by mixing equal volumes of each experimental sample and periodically inserted into the analysis sequence.

### 2.6. UHPLC-MS/MS Analysis

UHPLC-MS/MS analysis was performed using a Q Exactive™ HF mass spectrometer and a Vanquish UHPLC chromatograph (Thermo Fisher, Braunschweig, Germany). Separation was performed using a Hypesil Gold column (100 mm × 2.1 mm i.d., 1.9 μm) with a linear gradient program spanning 17 min at a constant flow of 0.2 mL/min. Two different mobile phase compositions were employed depending on the polarity mode. For the positive mode analysis, the gradient consisted of solvent A (water containing 0.1% formic acid) and solvent B (methanol). For negative polarity mode, eluent A was 5 mM ammonium acetate (pH 9.0) with methanol as eluent B. The solvent gradient was programmed as follows: an initial 2% of eluent B for 1.5 min, followed by a linear increase to 100% B over 12.0 min, maintained at 100% B until 14.0 min, then a linear return to 2% B over 14.1 min, and finally held at 2% B for 17 min (Table S1). Mass spectrometric analysis was performed using dual polarity detection with the following operational parameters: The scanning range was set to *m*/*z* 70–1050 Da. The electrospray ionization (ESI) source parameters were configured as follows: spray voltage, 3.2 kV; sheath gas flow rate, 40 arbitrary units; auxiliary gas flow rate, 10 arbitrary units; capillary temperature, 320 °C. Both positive and negative polarity modes were employed. MS/MS secondary scanning was performed in data dependent acquisition mode. It should be noted that the metabolomic profiles in this study represent the total culture activity. Due to the significant differences in biomass between the static and dynamic groups, the results were not normalized to cell number or protein content. Therefore, the observed changes reflect a combination of both increased biomass and potential metabolic shifts. Future studies employing per-cell normalization are required to further elucidate the intrinsic metabolic reprogramming of *A. muciniphila* at the individual cell level.

### 2.7. Metabolite Identification

Raw data underwent processing through Compound Discoverer 3.1 software (Thermo Fisher), adhering to the Metabolomics Standards Initiative (MSI) Level 2 confidence. To ensure precise identification, Peak alignment was conducted with strict criteria, including a retention time window of ±0.2 min and a mass deviation of 5 parts per million (PPM). The peak extraction protocol employed specific parameters: 5 PPM mass deviation tolerance, 30% acceptable signal intensity variation, minimum signal-to-noise ratio (SNR) of 3, and signal intensity threshold of 100,000. Measurements were conducted using both positive and negative ionization modes in separate analytical runs. The workflow included ion integration and molecular formula prediction based on both molecular and fragment ion peak patterns. Background ions were first eliminated by comparing samples with blank injections to ensure signal authenticity. To ensure data quality, features with missing values in more than 50% of the quality control (QC) samples were removed, and the remaining missing values were imputed using the k-nearest neighbor (KNN) method. Subsequently, total area normalization was performed to account for potential injection variance and to standardize the overall signal intensity across samples. The normalized data were then log-transformed and Pareto-scaled using MetaX software (v1.4.2, BGI-Shenzhen, Shenzhen, China) to reduce the influence of high-abundance metabolites. Finally, metabolite identification was achieved through spectral comparison across reference databases (mzCloud, mzVault, and Masslist) based on accurate mass and MS/MS fragment patterns.

### 2.8. Data Analysis

All experiments, including growth kinetics and SCFA analysis, were performed in triplicate (three independent biological runs, *n* = 3). Statistical analyses were conducted using Python (version 2.7.6), R (version 3.4.3), CentOS (release 6.6). Area normalization was applied to non-normally distributed data to ensure appropriate statistical processing. Metabolite identification was performed using three comprehensive databases: KEGG, HMDB, and LIPID Maps. Principal component analysis (PCA) was performed using MetaX software. Statistical significance was determined through *t*-tests. To address the multiple testing problem inherent in metabolomics, statistical significance was determined using the following criteria: Benjamini-Hochberg false discovery rate (FDR)-adjusted *p*-value (q-value) < 0.05, and fold change ≥ 2 or ≤0.5. The FDR correction was applied across all pairwise comparisons within each comparison group to minimize false-positive discoveries. Raw *p*-values from *t*-tests were adjusted using the p.adjust() function in R. Significant metabolic changes were visualized through volcano plots, which incorporated both magnitude of change (log_2_ fold change) and statistical significance (*p*-value). For the cluster heatmap, metabolite intensity was normalized using z-scores and plotted using the Pheatmap package in R. Metabolic pathway analysis was conducted using the KEGG database to explore metabolite functions and interactions. Pathway enrichment was considered significant if the number of differential metabolites (VIP > 1, q < 0.05, and FC ≥ 2 or ≤0.5) within the pathway exceeded a threshold (≥2 metabolites) and the FDR-adjusted *p*-value was <0.05.

## 3. Results and Discussion

### 3.1. Enhanced A. muciniphila Growth Under Dynamic Conditions

**Figure 2:**
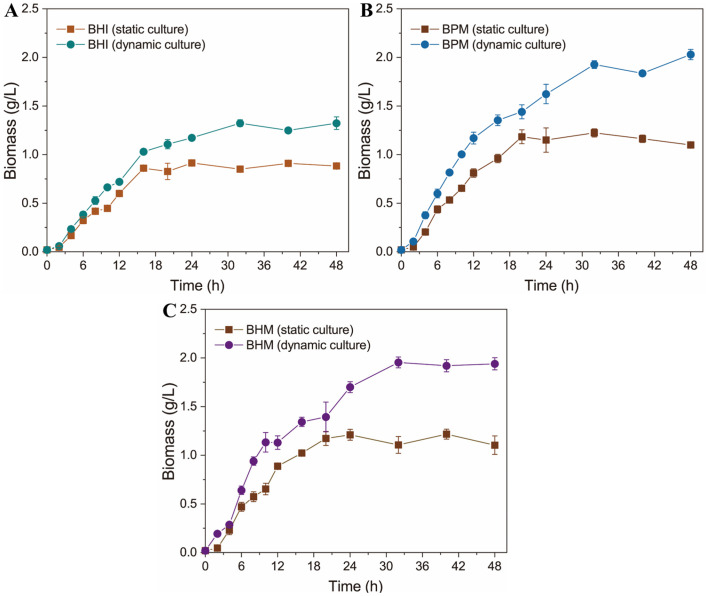
Growth curves of *A. muciniphila* under different culture conditions. The bacterium was cultured in (**A**) BHI, (**B**) BPM, and (**C**) BHM media under static or dynamic conditions. All growth experiments were performed with three independent biological replicates (*n* = 3). Data are presented as mean ± standard deviation (SD).

The human intestine exhibits peristaltic dynamics [27]. Dynamic cultivation conditions can expose intestinal microbiota to enriched nutrient profiles, thereby promoting growth performance [28]. Therefore, to better mimic the in vivo growth conditions of *A. muciniphila* within the human gastrointestinal tract and to optimize growth, we employed a gastrointestinal reactor system to dynamically simulate the human intestinal environment for *A. muciniphila*. The growth of *A. muciniphila* was analyzed under both static and dynamic conditions using BHI medium. The resulting growth curves revealed distinct patterns between static and dynamic cultures. Under static conditions, the biomass increased during the initial 16 h. Subsequently, due to nutrient depletion and metabolite accumulation in the vicinity of the strain, growth ceased, with the maximum biomass reaching 0.91 ± 0.02 g/L. Under dynamic conditions, the strain exhibited improved access to nutrients, resulting in a gradual increase in biomass over the first 32 h and reaching a maximum biomass of 1.32 ± 0.03 g/L, which was 45.05% higher than that of the static culture (Figure 2A). These results indicate that dynamic cultivation conditions significantly enhance the cell growth of *A. muciniphila*.

In the human intestinal tract, gut microorganisms utilize mucin as their primary carbon source and energy substrate. Derrien et al. [29] reported that the adding mucin to the culture medium significantly increases the biomass of *A. muciniphila*. Therefore, to improve the biomass of *A. muciniphila*, 2.5 g/L of porcine mucin was supplemented (BPM medium). As shown in Figure 2B, mucin supplementation significantly increased biomass under both static and dynamic conditions. Under static cultivation, the strain reached a maximum biomass at 18 h, achieving 1.18 ± 0.07 g/L. Subsequently, growth was inhibited by nutrient depletion and metabolite accumulation in the surrounding environment, with no further increase in biomass. During dynamic cultivation (0–48 h), the strain maintained a growth state throughout the period, reaching a maximum biomass of 2.03 ± 0.05 g/L, which was 72.03% higher than that of static cultivation using BPM medium and 123.08% higher than that of static cultivation using BHI medium. These results demonstrate that mucin can significantly enhance *A. muciniphila* biomass.

Although porcine mucin provides the carbon source, nitrogen source, and energy required for *A. muciniphila* growth, its O-glycan structures (such as sialylation and sulfation patterns) are similar to but exhibit species-specific differences from those of humans. Human mucin possesses human-specific glycosylation patterns, including complete human blood group antigens, which can more accurately simulate the human intestinal environment [30]. Therefore, we selected human mucin to replace porcine mucin to enhance the growth performance of *A. muciniphila*. Interestingly, human mucin supplementation (BHM medium) provided no additional growth benefits compared to BPM medium under the conditions tested (Figure 2C). This contrast with previous findings [25] may be due to the enhanced mass transfer efficiency of the current peristaltic design, which appears to reach a nutrient saturation threshold at 2.5 g/L regardless of mucin origin.

### 3.2. Comparison of SCFAs Production in Static and Dynamic A. muciniphila Cultures

**Figure 3:**
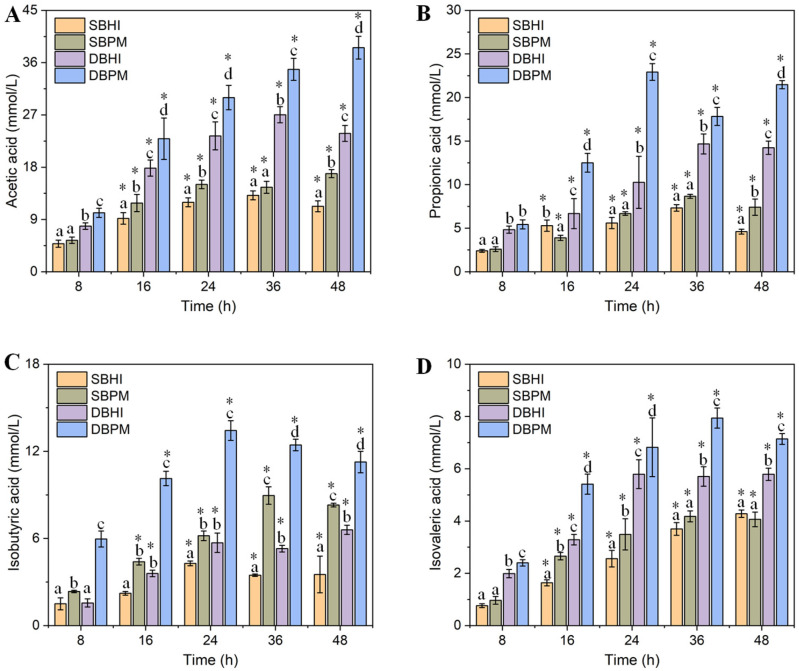
SCFAs produced by *A. muciniphila*, including acetic acid (**A**), propionic acid (**B**), isobutyric acid (**C**) and isovaleric acid (**D**) under different culture conditions. Statistical significance is denoted by differing letters in within-group (*p* < 0.05). The asterisk (*) indicates a statistically significant difference between the 8 h fermentation time and the other time points (16, 24, 36, and 48 h) (*p* < 0.05).

SCFAs, as the primary metabolites of *A. muciniphila*, play pivotal roles in neuro-immunoendocrine regulation [10,31,32]. In this study, we determined the concentration of SCFAs (including acetic acid, propionic acid, butyric acid, isobutyric acid, isovaleric acid and valeric acid) in BHI and BPM media under static and dynamic culture conditions, respectively. As shown in Figure 3A, compared to static conditions, dynamic culture significantly enhanced acetic acid production. Additionally, the addition of porcine mucin also significantly increased acetic acid concentrations. After 48 h of cultivation, the acetic acid concentration in dynamically cultured BPM medium (DBPM) peaked at 38.57 ± 1.96 mmol/L. This value was 3.43-, 2.28-, and 1.62-fold higher than those in statically cultured BHI (SBHI), statically cultured BPM (SBPM), and dynamically cultured BHI (DBHI) media, respectively. Acetic acid possesses multiple physiological functions including appetite suppression, energy metabolism regulation, intestinal inflammation inhibition, insulin sensitivity improvement, and lipid metabolism modulation [33,34,35].

The implementation of dynamic culture conditions combined with porcine mucin supplementation similarly enhanced propionic acid and isovaleric acid production (Figure 3B,D). Under DBPM conditions, the maximum propionic acid concentration reached 22.92 ± 0.96 mmol/L, representing 3.12-fold, 2.65-fold, and 1.56-fold increases compared to SBHI, SBPM, and DBHI conditions, respectively. Propionic acid plays a crucial role in glucose metabolism regulation and immune modulation [34,36]. Under DBPM conditions, the maximum isovaleric acid concentration reached 7.94 ± 0.38 mmol/L, showing 1.85-fold, 1.90-fold, and 1.37-fold increases compared to SBHI, SBPM, and DBHI conditions, respectively. However, under static culture conditions, porcine mucin supplementation did not enhance propionic acid and isovaleric acid production. We hypothesize that this may be attributed to inadequate contact between the supplemented porcine mucin and *A. muciniphila* under static conditions. Mucin, as a high-molecular-weight glycoprotein, may undergo gradual sedimentation in the absence of mixing, creating spatial heterogeneity that limits bacterial access to this substrate. Visual observation during our experiments revealed that static cultures developed visible stratification after 24 h, with a denser layer forming at the bottom of the culture vessel. While visual stratification was observed in static cultures, the role of substrate sedimentation in limiting metabolic productivity remains a hypothesis requiring further experimental validation.

Isobutyric acid synthesis is derived from protein degradation processes. Porcine mucin supplementation in the culture medium significantly increased isobutyric acid concentrations (Figure 3C). Furthermore, dynamic culture conditions further enhanced isobutyric acid production, with maximum concentrations reaching 13.43 ± 0.68 mmol/L, representing 3.14-fold, 1.50-fold, and 2.04-fold increases compared to SBHI, SBPM, and DBHI conditions, respectively. Isobutyric acid and isovaleric acid are both branched short-chain fatty acids (BSCFAs), which enhance the intestinal barrier and possess immune regulatory functions [37].

Regardless of cultivation conditions employed, butyric acid and valeric acid were not detected throughout the culture process. Butyric acid synthesis requires the key enzyme butyryl-CoA:acetate CoA-transferase, which is absent from *A. muciniphila*’s metabolic pathways. Despite the inability to directly produce butyrate, the implications for *A. muciniphila’s* probiotic functionality remain profoundly positive. The primary probiotic effects of this strain are largely mediated by its high-yield production of acetate and propionate. These abundant SCFAs directly contribute to physiological benefits such as appetite suppression, improved insulin sensitivity, and immune modulation. Furthermore, when *A. muciniphila* is co-cultured with other intestinal microorganisms (such as *Anaerostipes caccae*), these commensals can incorporate the massive amounts of *A. muciniphila*-derived acetate through metabolic cross-feeding to synthesize butyrate [38]. Thus, the probiotic functionality of *A. muciniphila* involves not only the direct action of acetate and propionate but also the indirect enhancement of the gut butyrate pool and overall microbial homeostasis through synergistic interactions [39,40]. Valeric acid can be produced through metabolic pathways involving proline or odd-chain fatty acids. Since the culture medium contained only minimal amounts of proline or odd-chain fatty acids, insufficient substrate availability prevented *A. muciniphila* from producing detectable valeric acid concentrations.

To determine whether enhanced SCFA production under dynamic conditions represents a true metabolic shift or simply reflects increased biomass, we calculated an approximate yield metric (q_SCFA_, mmol/g DCW) by normalizing total SCFA (acetic acid, propionic acid, isobutyric acid, and isovaleric acid) production to final biomass (Table 1). These data reveal that dynamic cultivation increases specific SCFA productivity compared to static conditions within the same medium type (DBHI vs. SBHI: 1.41-fold; DBPM vs. SBPM: 1.26-fold). The enhanced SCFA titers under dynamic conditions might result from improved mass transfer. Peristaltic motion may enhance metabolic flux through SCFA biosynthetic pathways, possibly through improved mass transfer of substrates and removal of inhibitory metabolites at the cellular level.

### 3.3. Global Metabolites Change Under Different Cultured Conditions

#### 3.3.1. QC and Samples Characteristics

**Figure 4:**
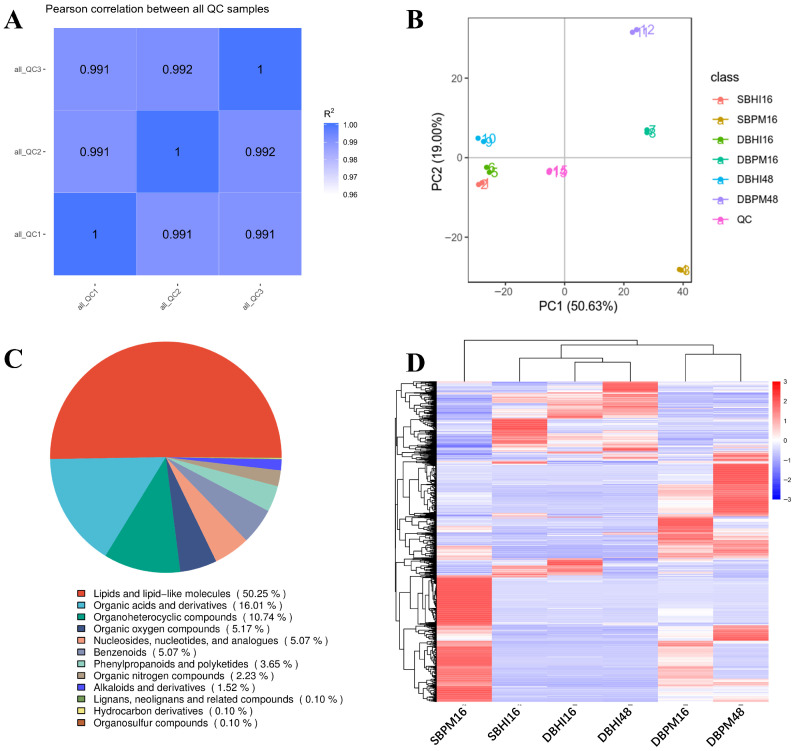
Analysis of differential metabolite expression in *A. muciniphila* under different culture conditions. (**A**) Analysis of QC sample correlation; (**B**) Analysis of Principal Component (PCA) of all samples; (**C**) Differential metabolite classification ring map (HMDB); (**D**) Differential metabolite hierarchical clustering, with red indicating greater relative expression levels and blue indicating fewer relative expression levels. The figure shows the metabolite classification information based on the KEGG and HMDB databases. The sample nomenclature denotes the culture method and duration: labels beginning with ‘S’ (e.g., SBHI16, SBPM16) correspond to static culture, while those prefixed with ‘D’ (e.g., DBHI16, DBPM16, DBHI48, DBPM48) represent dynamic culture. All samples were cultivated in either BHI or BPM medium for 16 or 48 h, as indicated by the suffix.

To understand the metabolic responses under different culture conditions, we analyzed metabolites generated by *A. muciniphila* using UHPLC-MS/MS untargeted metabolomics. It should be noted that the metabolomic analysis was conducted on an asymmetric schedule (Static: 16 h; Dynamic: 16 h and 48 h). This design was chosen because the static culture reaches its stationary and decline phase early (around 16 h), making late-stage (48 h) comparisons physiologically irrelevant. To ensure clarity, the 16 h samples were used to compare the effects of culture mode (static vs. dynamic) at a matched time point, while the 48 h dynamic samples were used to assess the metabolic shifts during extended cultivation in the bioreactor. A portion of each sample was used to create a QC sample, which was placed in the sample detection queue to assess system stability and data quality throughout the experiment. The QC sample was scanned in segments after completion of the sample detection, along with secondary spectra from the experimental samples, for metabolite characterization [41]. The correlation analysis of QC samples demonstrated excellent analytical reproducibility throughout the metabolomics experiment (Figure 4A). Pearson correlation coefficients among all QC samples exceeded 0.991. This high degree of correlation indicates minimal instrumental drift and batch effects during the analytical process, ensuring that the observed metabolic differences between experimental groups reflect true biological variation rather than technical artifacts. The tight clustering of QC samples also confirms that sample preparation, chromatographic separation, and mass spectrometric detection remained consistent throughout the analysis, validating the reliability of the subsequent comparative metabolomics analysis.

Principal component analysis (PCA) was employed to visualize the global metabolic variation among *A. muciniphila* samples cultured under different conditions (Figure 4B). The first two principal components captured 69.63% of the total metabolic variance, with PC1 and PC2 accounting for 50.63% and 19.00%, respectively. The 3D PCA plot showed that the explained variance for the third component (PC3) was 9.9% (Figure S1). The PCA score plot revealed clear separation among experimental groups, including static culture with BHI for 16 h (SBHI16), static culture with BPM for 16 h (SBPM16), dynamic culture with BHI for 16 h and 48 h (DBHI16, DBHI48), and dynamic culture with BPM for 16 h and 48 h (DBPM16, DBPM48). The QC samples clustered tightly in the positive PC1 region, demonstrating excellent analytical reproducibility and confirming the reliability of the metabolomics data throughout the experimental process. PC1, which explained the majority of variance (50.63%), effectively separated samples based on the combined effects of culture method and medium composition. Static culture samples (SBHI16 and SBPM16) were positioned in the negative PC1 region, while dynamic culture samples showed greater dispersion along the PC1 axis, suggesting that dynamic cultivation induces more pronounced metabolic changes compared to static culture. PC2 (19.00% variance) captured the metabolic differences associated with medium composition and cultivation time. Notably, BPM-cultured samples (SBPM16, DBPM16, DBPM48) showed distinct separation from BHI-cultured samples along the PC2 axis, indicating that mucin supplementation substantially alters the metabolic landscape of *A. muciniphila*.

#### 3.3.2. Screening of Differential Metabolites

The classification of all 1463 annotated metabolites according to the HMDB database (Figure 4C) revealed that lipids and lipid-like molecules constituted the largest proportion (50.25%), followed by organic acids and derivatives (16.01%), organoheterocyclic compounds (10.74%), organic oxygen compounds (5.17%), nucleosides and nucleotides (5.07%), and benzenoids (5.07%). The predominance of lipids in the metabolome is consistent with the known role of *A. muciniphila* in membrane lipid metabolism and its interaction with the intestinal mucus layer. The substantial proportion of organic acids reflects the fermentation activity of this bacterium, particularly the production of short-chain fatty acids.

Differential metabolites were screened with VIP > 1, FDR-adjusted *p*-value (q-value) < 0.05, and FC ≥ 2 or ≤0.5. A total of 1294 differential metabolites were identified, with 738 and 556 significant features detected in positive and negative ion modes, respectively. The differential metabolites of *A. muciniphila* under varying culture conditions were further evaluated using hierarchical cluster analysis (HCA) (Figure 4D). The heat map visualized sample clustering along the horizontal axis and metabolite abundance along the vertical axis, with dendrogram branch lengths indicating the degree of similarity in metabolite composition among groups. The color intensity gradient from blue to red represents increasing metabolite abundance. As shown in Figure 4D, both the culture medium (BHI versus BPM) and the cultivation conditions (static versus dynamic) significantly modulated the metabolite profiles of *A. muciniphila*. These results indicate that the metabolic activity of the strain is significantly influenced by these two variables.

#### 3.3.3. Analysis of Differential Metabolites

**Figure 5:**
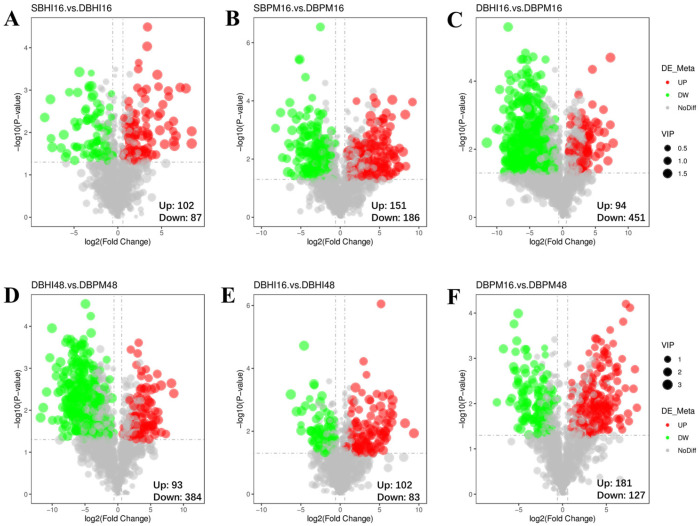
Volcano plots depicting differential metabolites across comparative groups: (**A**) SBHI16 vs. DBHI16; (**B**) SBPM16 vs. DBPM16; (**C**) DBHI16 vs. DBPM16; (**D**) DBHI48 vs. DBPM48; (**E**) DBHI16 vs. DBHI48; (**F**) DBPM16 vs. DBPM48. The sample nomenclature denotes the culture method and duration: labels beginning with ‘S’ (e.g., SBHI16, SBPM16) correspond to static culture, while those prefixed with ‘D’ (e.g., DBHI16, DBPM16, DBHI48, DBPM48) represent dynamic culture. All samples were cultivated in either BHI or BPM medium for 16 or 48 h, as indicated by the suffix. The horizontal axis represents the log_2_-transformed fold change of metabolites, while the vertical axis corresponds to the negative logarithm (base 10) of the *p*-value. Each point denotes a metabolite, with red and green colors indicating significantly up- and down-regulated metabolites, respectively. Dot sizes reflect the variable importance in projection (VIP) scores.

The differential metabolites identified across various comparative groups were visualized using volcano plots. Figure 5A showed the volcano plot of differential metabolites between SBHI16 and DBHI16, in which 102 metabolites were upregulated and 87 were downregulated. Among these, 2-hydroxycinnamic acid exhibited the most pronounced upregulation, with an 80.26-fold increase under the DBHI16 condition relative to SBHI16. It is noteworthy that 2-hydroxycinnamic acid is a bioactive compound with antioxidant properties, which contributes to the protection of cellular membranes, proteins, and DNA against oxidative damage [42]. Figure 5B displayed the volcano plot of differential metabolites between SBPM16 and DBPM16, showing 151 upregulated and 186 downregulated metabolites. The most significantly altered metabolites were lysophosphatidylinositol species, specifically LPI 18:2 and LPI 20:4, which were upregulated by 581.51-fold and 327.42-fold, respectively, under the DBPM16 condition compared to SBPM16. Figure 4D and Figure 5C both showed volcano plots of differential metabolites between BHI and BPM under dynamic culture conditions. A notable change was observed in hydrocinnamic acid, which was markedly upregulated by 155.21-fold and 159.91-fold at 16 h and 48 h, respectively, when BPM medium was used compared to BHI. The increase in hydrocinnamic acid might enhance the host’s resistance to bacterial infections and oxidative stress mediated by reactive oxygen species [43]. Figure 5E showed the volcano plot of differential metabolites between DBHI16 and DBHI48, indicating 102 upregulated and 83 downregulated metabolites. With prolonged fermentation time, organic acids such as glutamic acid, and 2-isopropylmalic acid were significantly increased. Figure 5F showed the volcano plot of differential metabolites between DBPM16 and DBPM48, with 181 metabolites upregulated and 127 downregulated. Extended fermentation led to a notable increase in lipid species, primarily phosphatidylcholines (PC) and phosphatidic acids (PA). Extended fermentation led to a notable increase in lipid species, primarily PC and PA. The detected PC mainly originated from intracellular membrane turnover of *A. muciniphila* rather than secreted metabolites [44]. Follow-up experiments with separate extraction of intracellular and extracellular metabolites are needed to quantify the secretion ratio.

While untargeted profiling revealed significant shifts in several metabolites with high fold changes, it should be noted that these represent putative identifications based on library matching. Future studies employing synthetic standards for absolute quantification are needed to confirm the precise intracellular concentrations of these specific biomarkers.

#### 3.3.4. Analysis of Differential Metabolic Pathway

Metabolic pathway analysis is an effective approach for elucidating the direct connections among metabolites and reconstructing biochemical reaction networks [45]. To identify the metabolic pathways potentially associated with *A. muciniphila* cultured under different cultivation conditions, all differential metabolites were subjected to pathway enrichment analysis using MetaboAnalyst 5.0. Pathway enrichment was performed using the hypergeometric test (over-representation analysis) with the KEGG pathway library as the reference background. Pathway enrichment was considered significant if the number of differential metabolites (VIP > 1, q < 0.05, and FC ≥ 2 or ≤0.5) within the pathway exceeded a threshold (≥2 metabolites) and the FDR-adjusted *p*-value was <0.05. The top 10 most significantly enriched metabolic pathways primarily encompassed amino acid biosynthesis, purine metabolism, fatty acid biosynthesis, and pyrimidine metabolism.

**Figure 6:**
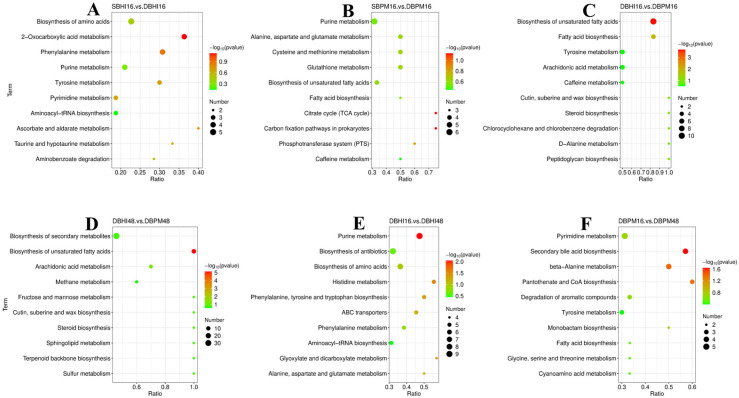
Metabolite pathway enrichment analysis plot of metabolite biomarkers across comparative groups: (**A**) SBHI16 vs. DBHI16; (**B**) SBPM16 vs. DBPM16; (**C**) DBHI16 vs. DBPM16; (**D**) DBHI48 vs. DBPM48; (**E**) DBHI16 vs. DBHI48; (**F**) DBPM16 vs. DBPM48. The sample nomenclature denotes the culture method and duration: labels beginning with ‘S’ (e.g., SBHI16, SBPM16) correspond to static culture, while those prefixed with ‘D’ (e.g., DBHI16, DBPM16, DBHI48, DBPM48) represent dynamic culture.

Under dynamic culture conditions, significant alterations were observed in pathways associated with amino acid synthesis and metabolism (Figure 6A,B). Amino acids serve as the fundamental building blocks for all enzymatic and structural proteins. In the absence of amino acids, ribosomes cannot synthesize proteins, leading to an immediate cessation of cellular growth and division [46]. When carbon or energy sources are limited, the strain can decompose amino acids via deamination, generating carbon skeletons that support bacterial growth [47]. Proline metabolism was also significantly upregulated. Proline functions as a compatible solute that helps maintain cellular water content. We hypothesize that proline may act as a compatible solute or energy source to maintain redox balance, which requires further verification by targeted enzyme activity assays [48,49]. However, we did not directly measure intracellular redox status or proline dehydrogenase activity. This mechanistic interpretation therefore remains speculative and requires validation through targeted enzymatic assays and metabolic flux analysis.

Purine and pyrimidine metabolism pathways showed significant changed across multiple comparison groups (Figure 6A,B,E,F), indicating active nucleotide turnover under different culture conditions. Purine metabolism was particularly prominent in the SBPM16 vs. DBPM16 comparison (Figure 6B) and DBHI16 vs. DBHI48 comparison (Figure 6E), suggesting that dynamic cultivation and extended fermentation time stimulate nucleotide biosynthesis. Nucleotides serve as essential building blocks for DNA and RNA synthesis, and their increased production might reflect enhanced cellular proliferation under dynamic culture conditions. Furthermore, purine derivatives such as adenosine and inosine have been reported to exert immunomodulatory effects in the gut [50], which might contribute to the probiotic functionality of *A. muciniphila*. Pyrimidine metabolism was notably enriched in the DBPM16 vs. DBPM48 comparison (Figure 6F), indicating that extended cultivation in mucin-containing medium might promote pyrimidine nucleotide synthesis, potentially supporting increased bacterial replication.

**Figure 7:**
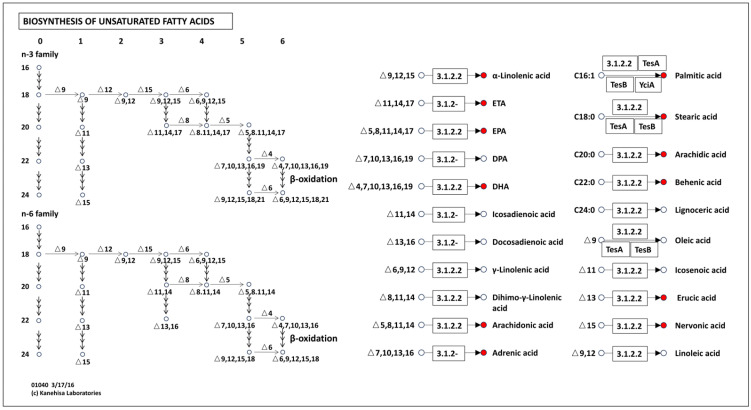
Unsaturated fatty acid synthesis pathway. Differential metabolites in each pathway are indicated in red dot.

The fatty acid biosynthetic pathways, especially the unsaturated fatty acid synthesis pathway, exhibited significant changes in *A. muciniphila* when cultured in BPM medium compared with BHI medium (Figure 6C,D). Fatty acids are critical components of the cell membrane. Under environmental stress conditions, microorganisms can mitigate stress-induced injury by adjusting the fatty acid composition of their cell membranes [51]. Cultivation in BPM medium resulted in a marked decrease in unsaturated fatty acid synthesis (Figure 7). Based on established membrane biology principles, reduced unsaturated fatty acid content typically decreases membrane fluidity [52]. Altered fatty acid profile may represent a membrane adaptation response, though the functional consequences for stress tolerance require experimental validation through membrane characterization studies.

The enhanced production of bioactive metabolites under dynamic cultivation conditions has important implications for pharmaceutical development. The substantial increases in short-chain fatty acids (acetic acid: 38.57 ± 1.96 mmol/L; propionic acid: 22.92 ± 0.96 mmol/L) are particularly relevant, as SCFAs are recognized therapeutic agents for maintaining gut health, modulating immune responses, and regulating metabolic processes [53]. The 80.26-fold upregulation of 2-hydroxycinnamic acid, a compound with documented antioxidant and anti-inflammatory properties, suggests that dynamically cultivated *A. muciniphila* may possess enhanced therapeutic potential compared to statically cultured bacteria. Furthermore, the increased production of phosphatidylcholines and other lipid species indicates improved membrane composition, which may enhance bacterial viability during downstream processing, storage, and gastrointestinal transit. These metabolic enhancements could support the development of more effective probiotic formulations and postbiotic products.

The increase in SCFAs under dynamic culture is consistent with metabolomic results showing upregulation of amino acid metabolism, pyruvate metabolism, and fatty acid degradation pathways. These pathways provide sufficient precursors and energy for SCFA synthesis, forming a coordinated metabolic network that supports both growth and functional metabolite production. Furthermore, it is important to acknowledge that this study analyzed the whole culture metabolome, which conflates intracellular metabolites, secreted molecules, and residual media components. While this provides a holistic ‘metabolic footprint’ of *A. muciniphila* in a simulated gastrointestinal environment, it limits our ability to definitively attribute these changes to specific intracellular flux or extracellular secretion. Future studies utilizing separate extraction protocols for cell pellets and supernatants are warranted to further decouple these metabolic compartments.

## 4. Conclusions

This study represents the investigation of the cultivation of *A. muciniphila* using an improved gastrointestinal reactor that simulates intestinal peristalsis, providing crucial insights into the dynamic growth behavior and metabolic characteristics of this important next-generation probiotic. Dynamic cultivation achieved remarkable improvements in bacterial biomass, with increases of 45.05% in BHI medium and 72.03% in mucin-supplemented BPM medium compared to traditional static culture, reaching a maximum biomass of 2.03 ± 0.05 g/L. The production of health-promoting short-chain fatty acids was significantly enhanced, with acetic acid reaching 38.57 ± 1.96 mmol/L and propionic acid achieving 22.92 ± 0.96 mmol/L, representing substantial multi-fold increases with important implications for metabolic regulation, immune modulation, and intestinal barrier function. The metabolomic data showed significant differences in 1294 metabolites. Dynamic cultivation induced substantial upregulation of bioactive compounds including 2-hydroxycinnamic acid (80.26-fold increase) and phosphatidylcholine species, while pathway enrichment analysis demonstrated significant alterations in amino acid biosynthesis, fatty acid metabolism, and nucleotide metabolism. These findings establish a foundation for optimizing large-scale *A. muciniphila* production for commercial probiotic applications, as the enhanced biomass and beneficial metabolite synthesis could significantly improve production efficiency and therapeutic efficacy.

## Figures and Tables

**Table 1:** Approximate yield metric under different culture conditions in 48 h.

Condition	Biomass (g/L)	Total SCFA (mmol/L)	q_SCFA_ (mmol/g DCW)
SBHI	0.88 ± 0.02	23.67 ± 0.65	26.93 ± 0.78
SBPM	1.10 ± 0.01	33.67 ± 0.51	30.61 ± 0.48
DBHI	1.32 ± 0.06	50.43 ± 0.67	38.21 ± 1.83
DBPM	2.03 ± 0.05	78.45 ± 0.83	38.65 ± 1.05

All experiments were performed with three independent biological replicates (*n* = 3). Note: q_SCFA_ represents an approximate yield metric based on final biomass, and may not fully reflect instantaneous per-cell metabolic rates across the entire growth curve.

## Data Availability

The original contributions presented in the study are included in the article, further inquiries can be directed to the corresponding author.

## Supplementary Materials

The following supporting information can be downloaded at: https://www.mdpi.com/article/10.3390/foods15091467/s1, Figure S1: Analysis of 3D PCA plot of all samples; Table S1: UHPLC gradient elution program of negative polarity mode; Table S2: The differential metabolites identified across various comparative groups.
