# Supplementary material for: Dynamic Cultivation of Akkermansia muciniphila in an Improved Gastrointestinal Reactor: Enhanced Growth and Metabolomic Profiling
Source: Foods. 2026 Apr 22;15(9):1467. doi: 10.3390/foods15091467 (PMC13164191; doi:10.3390/foods15091467)
Supplement: Supplementary file 1 [file foods-15-01467-s001.zip › foods-4253752-supplementary.pdf]

Dynamic cultivation of *Akkermansia muciniphila* in an improved gastrointestinal reactor: enhanced growth and metabolomic profiling

Yuqin Wang [1†], Kexin Yu [1†], Tongyan Shen [1], Kunqing Huang [1], , Mengdie Li [1], Yating Wang [1], Jiaqi Xi [1], Jintian Chen [1], Minjie Gao [2], Zhitao Li [1,2 *]

[1] School of Grain Science and Technology, Jiangsu University of Science and Technology, Zhenjiang 212100, China

[2] State Key Laboratory of Food Science and Resources, Jiangnan University, Wuxi, Jiangsu 214122, China

*Corresponding authors:

E-mail address: lizhitao@just.edu.cn (Z.T. Li)

[†] These authors contributed equally to this work.

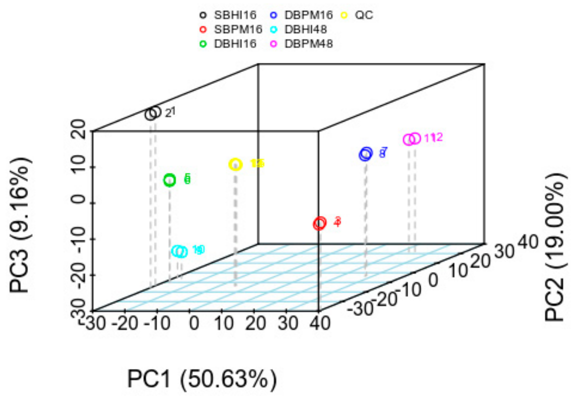

Figure S1. Analysis of 3D PCA plot of all samples

Table S1. UHPLC gradient elution program of negative polarity mode.

Time (min)	Solvent A (%)	Solvent B (%)
0-1.5	98	2
1.5-13.5	98→0 (Linear)	2→100 (Linear)
1.35-14.0	0	100
14.0-14.1	0→98 (Linear)	100→2 (Linear)
14.1-31.1	98	2

Table S2 The differential metabolites identified across various comparative groups.

Num.	Name	SBHI16	SBPM16	DBHI16	DBHI48	DBPM16	DBPM48
1	Acetophenone	94181620965	1284153302	142023243823	135377200202	9657728233	12634316588
2	Indole-3-acrylic acid	55918294872	13876926108	988047995	904830916	168249721	291779690
3	3-Phenyllactic acid	21012714	46510843	121140166	269429807	62135486	141975477
4	OxPC (16:0-18:0+2O)	132625	58900036	199194	200852	26567130	750632
5	LPC 17:0	918151	845360160	3377954	972832	159059110	1662821
6	Prostaglandin F3α	437665	28019324	495904	377214	29333170	58378264
7	LPC 15:0	3803022	3293463671	17726864	1482396	299899336	7576484
8	methyl 3,4,5-trihydroxycyclohex-1-ene-1-carboxylate	37863072	263727912	4582333	4170427	15629055	19834029
9	Glycerol-3-phosphate	16113764	24097163	13240323	9288329	36817672	35647548
10	Caprolactam	18523144	77767624	116748977	316479024	1541898589	4241163107
11	Puromycin	1413824	6556365	1191996	1002741	33617109	63674882
12	Gamma-Caprolactone	22460577	33642311	72674172	57190570	35602958	60883949
13	PC (14:0e/5:0)	749735	258428818	1410916	1428496	217944269	24872691
14	LQH	61834110	2885709	5052995	26876248	3677412	8819414
15	Isobutyrylglycine	57241198	13049688	48694868	51563218	33477180	49806904
16	3,8,9-trihydroxy-10-propyl-3,4,5,8,9,10-hexahydro-2H-oxecin-2-one	16297818	40967747	3714514	1815069	29333303	1937220
17	2-(2-amino-3-methylbutanamido)-3-phenylpropanoic acid	41383159	20265734	25460371	70000293	16796517	61718702
18	(3beta,9xi)-3-(beta-D-Glucopyranosyloxy)-14-hydroxycard-20(22)-enolide	7567514	770163	9068888	17641937	2281941	2481313
19	DL-Carnitine	787146949	160125229	536147782	644099208	472062865	339945793
20	Oleamide	8527864	12679539	2483220	2449588	11247882	286512733

21	OxPC (18:1-18:3+1O)	202293	52365931	560716	242945	19221110	553349
22	Oleoyl-L-α-lysophosphatidic acid	15204615	6746238159	54145033	8770519	243861854	264858445
23	3-Cresotinic acid	12736481	27389044	7945804	7461524	26014157	59770476
24	PE (2:0/16:2)	3669117	3113225334	4962637	1878884	192506282	18162619
25	Sphingosine (d18:1)	6185901	3314135462	10336290	7177217	1915745558	2416193295
26	3-Hydroxylidocaine	59161032	4724829	25209908	38680722	19135769	24300116
27	23-Nordeoxycholic acid	155218	7275267	123429	113766	60519912	28561647
28	APK	56204171	5309106	5210526	8214282	2986263	3283210
29	Ala-Ile	58496676	41269028	5655926	16081764	9089420	8624314
30	N-Tetradecanamide	13010235	230530047	11286946	17378201	182996835	162934227
31	Pantethine	1288045	360865	13088288	14010436	598920	476191
32	N-Acetyl-L-tyrosine	5190150	1053040	14282577	14039719	2151006	4522887
33	1-Methyladenine	59227277	26943737	20744100	5848745	23921347	55262612
34	Leucine-enkephalin	19085560	7169344	13400616	68435039	11148753	3363497
35	13,14-dihydro-15-keto-tetranor Prostaglandin D2	693912	1418583	558883	615616	20038715	55468514
36	N-(4-chlorophenethyl)-1-adamantanecarboxamide	218594832	16771082	182517571	50139363	10454327	15628630
37	Desthiobiotin	320931251	49050831	770502283	941333887	453680767	688438037
38	Agmatine	31589435	3044393709	66793667	2963113	27663391	50210725
39	Milbemycin A3 oxime	3501788	2256760	1563658	72467206	2255219	1423823
40	Leucine enkephalin amide	15239091	757781	13594580	11485253	3018264	1690892
41	LPS 20:1	250237	53065751	252221	153217	26745709	4144990
42	5α-Tetrahydrocortisol	3735271	248387912	2216352	2390716	115709648	229295049
43	JWH 250 N-pentanoic acid metabolite	58500852	11760327	27927451	41386223	14302405	17359691
44	1-Oleoyl-Sn-Glycero-3-Phosphocholine	3504054	800558566	9193864	1961759	96041792	15526243
45	KNH	723499994	20922671	576039739	122529124	384381451	58566365

46	FNK	47545942	5367062	50673521	2998663	5946295	3740119
47	PC (7:0/8:0)	7021862	203247943	12550795	13049515	139459175	295944640
48	PC (17:0/22:5)	156661	53928281	134870	130643	28597674	718585
49	ethyl 3-oxo-3-(1H-pyrazol-5-ylamino)propanoate	30876155	2004307	48150343	73909738	2051927	2174521
50	PE (5:0/13:1)	3889097	2634168156	3655482	2347562	94685106	28000340
51	Glycoursodeoxycholic acid	367850	34475857	247884	1526636	17009278	53230538
52	DL-Tryptophan	55503900773	13807889949	1019015869	920030214	666994447	405822802
53	UMP	1078792	44796665	3954650	1047765	8209668	2423384
54	PE (2:0/18:2)	97254	51508950	87697	85824	921999	663316
55	Taurochenodeoxycholic acid	215305	49045330	467675	167975	4105987	4276529
56	3-(3-methylbut-2-en-1-yl)-3H-purin-6-amine	54572959	17851303	41074124	11293827	44085505	206005600
57	LPE 20:3	377641	797480326	1391639	423611	211778781	5266515
58	Hyodeoxycholic acid	95709167	488861432	60955483	38255682	3509817455	390604248
59	Sulfamerazine	34976703	8525116	57800263	60971792	14773009	24478494
60	LPA 16:1	14231844	22675704	5245673	1459298	10326577	5616101
61	5-Methylcytosine	2232135	227505175	2543399	2980645	34177686	33951200
62	L-Glutamic acid	144484066	13258009	208439177	7015496	13292600	17939341
63	Caffeine	80887993	35056227	214826488	198778818	57335531	55060067
64	LPE 22:1	648692	225915861	797977	912897	171689980	33580490
65	4-Acetamidobutanoic acid	9580679	27325198	9832557	211443037	211816376	176946344
66	Estra-1(10),2,4,16-tetraen-3-ol	14201336	525259	7327673	5866788	1911166	1370277
67	Palmitoylcarnitine	537344	228825768	533306	441831	18047830	2768473
68	YLK	3851107	219277422	4180238	5120783	19382872	4072318
69	N-(5-acetamidopentyl)acetamide	8900860	120277648	794942198	792962962	9633022	14900155
70	OxPC (18:1-18:2+2O)	179915	47720668	171765	200983	26313692	569479
71	Arachidonoyl ethanolamide phosphate	671734115	2843796	4806863	4110682	2468878	3370667

72	γ-Linolenic acid ethyl ester	1826242	224487975	3384542	5774415	54858607	23022350
73	LPE 22:4	651150	748181943	6081405	775643	178656784	5180224
74	LPG 17:1	316239	47401181	5742310	820758	4342252	1365238
75	PEtOH (18:1-18:2)	167932	19006878	148370	150981	39296740	41452310
76	Hexanoylglycine	13693080	9878895	212509107	100004909	9713358	10585361
77	Taurodeoxycholic Acid	700741599	7400572	157068095	217811046	226900285	6596191
78	PE (5:0/16:0)	189074	44448112	118581	86117	4179936	390591
79	N6-Acetyl-L-lysine	60876361	32111124	209153079	720502200	84115081	1560776012
80	OxPE (16:0-18:0+1O(1Cyc))	137532	40584008	134293	132841	48830189	2956187
81	3-(4-methoxyphenyl)-1-methyl-1H-1,2,4-triazol-5-ol	3093001	195402358	2757397	4220649	24540202	28164559
82	2–Furanoic Acid	2862470	4489101	10562000	13094938	19971801	12871862
83	3-O-β-D-Glucopyranosylandrographolide	61562571	6268879	311843479	827820366	20375753	2882834
84	D-α-Hydroxyglutaric acid	12251230	6709205	4522330	1312791	23161082	25529631
85	(5Z)-3-aminonon-5-enoic acid	4352156	206428737	5582133	2480484	20213660	26853707
86	LPC 18:2	7881945	6184440777	39263978	2231020	114500731	17355928
87	PC (14:0e/2:0)	29629584	18659555942	181180236	14109459	562520725	56767422
88	Cafestol	2877009	206675654	2658389	4045050	137276490	232394760
89	Ethyl-β-D-glucuronide	78815299	78342196	184291813	5708772	90161881	31881348
90	OxPC (18:0-18:2+2O)	134783	40185358	132953	130186	47519291	478236
91	(2E)-4-Hydroxy-3,7-dimethyl-2,6-octadien-1-yl beta-D-glucopyranoside	61042466	20846984	24767552	46639954	149675877	712827577
92	PC (16:0e/2:0)	327367	42943550	549720	286936	16956299	2635670
93	N'2-(2-furylcarbonyl)-3-chloro-4-methylthiophene-2-carbohydrazide	569866	1416254	1057214	13300896	3064551	3180644
94	19-Nortestosterone	50111882	37975680	46428471	52853657	49806664	152920139

95	2-ethyl-2-(3-methoxyphenyl)cyclohexanone oxime	1452218	4709563	808664	62362378	3356238	5786152
96	α-Lapachone	31929802	6658150	53004764	54732635	12944731	19730383
97	Deoxyadenosine	35207680	35031407	48081595	1485097	13281143	20483923
98	LPE 20:2	702537	211840924	1240463	945440	154168741	8419128
99	1,2,3-Benzenetriol	449672	1607429	2830392	10376317	12439637	10635048
100	Enterodiol	6675514	2197198	5281555	7307272	46369109	2197740
101	Monoolein	9359683	2287319099	44942295	32248330	2884556420	21510723334
102	Taurolithocholic acid sodium salt	210396	527323904	393535	123338	6978385	6262964
103	(12Z)-9,10,11-trihydroxyoctadec-12-enoic acid	60185298	244919230	42127807	39271932	667814476	3459868347
104	L-Ascorbate	874581	1027522	5037218	11825814	3149852	3588977
105	16α-Hydroxyestrone	9164275	27525027	1820459	2200151	19903508	41876787
106	1-acetyl-N-(4-ethoxyphenyl)-4-piperidinecarboxamide	12586697	617533	3915250	4058320	836958	1035278
107	N-lactoyl-phenylalanine	11927286	774352	9669285	9627249	833922	6910338
108	DL-2-(acetylamino)-3-phenylpropanoic acid	17661764	7748671	56270605	37728537	22383992	57594815
109	5-[(10Z)-14-(3,5-dihydroxyphenyl)tetradec-10-en-1-yl]benzene-1,3-diol	14823151	307580392	27323621	22359307	3023670709	338522999
110	MGDG (16:0/22:6)	197627	44720941	191672	204873	15495944	906491
111	N-Acetylmethionine	48298638	15169944	38617318	33337554	10074535	13676864
112	N-Acetylanthranilic acid	897915	2389416	1474363	12698253	1438687	16037754
113	2-Ketohexanoic acid	17311257	35229993	44826216	211724543	35080152	34648296
114	Norharman	47406096	103809565	11977320	12250992	116067601	141670887
115	Anserine	11929971	7310205	9556179	5056292	6307505	5021293
116	Arachidonic acid	5238916	5755218028	4171060	4118494	1717697230	1342277420
117	4-(3,4-dihydro-2H-1,5-benzodioxepin-7-ylamino)-4-oxobutanoic acid	19822119	16230033	20609697	58049560	37867782	111102676

118	L-Glutamine	8837510	9894588	12279343	4156938	7532480	9130690
119	4-oxo-4-[(1-phenylethyl)amino]but-2-enoic acid	46867001	10051041	43222498	57454284	20124964	36599875
120	Cytosine	21798254	2605887804	21416812	16718852	247718259	225595877
121	7-Methylxanthine	7989868	37989170	14251764	83044268	3140243377	4536311431
122	5-ethoxy-2-[(2,3,4,5,6-pentafluorobenzyl)thio]-1H-benzo[d]imidazole	751730	4423252	23566970	741007195	1312030324	377993965
123	Pyrogallol	1387301	3553653	4087202	6528193	18256515	34176798
124	WLK	41971387	2644762	3874614	919330	1173633	1464475
125	Diethyleneglycol diacetate	4698275	9361591	4858089	5298646	10864316	236321787
126	13,14-Dihydro-15-keto Prostaglandin A2	1852747	6605468	1792323	1718339	11139560	42402277
127	DLK	606912108	82875911	145559353	218722875	11726811	24786148
128	Indole-3-lactic acid	846352068	2978574	1588648862	1388073217	563518917	854579172
129	7-Ketolithocholic acid	1205429	3559998	1822782	1220030	17715929	3197678761
130	4-Oxoretinol	45462897	23945436	41680021	42617577	20705078	1718137
131	D-Sedoheptulose 7-phosphate	5708617	1078930	6699681	9073184	7507711	10423133
132	4-Pyridoxic acid	11411954	2844803	11179045	921992	3078047	3553217
133	Docosahexaenoic Acid	1645839	40777963	924452	683588	29823271	24262969
134	Tyrosol	6220367	10663814	5496610	11051748	10750306	44380087
135	trans-10-Heptadecenoic Acid	1556462598	954940082	651399470	197793028	1134514897	702635329
136	Asp-Phe methyl ester	38291537	49742038	45432970	202530674	76426701	100035233
137	Cadaverine	44881082	135926601	33193991	39402202	96154726	75769429
138	Maltopentaose	2737414	183831049	1653774	1616089	4130940	4966855
139	4-styrylpyridine	42210229	15967680	37958626	32741496	50895009	51849714
140	OxPS (18:0-18:1+3O)	209975	22733560	205332	191528	38417125	1397511
141	LPC 18:0	10662250	5551766105	32603527	17262181	2749676056	93308966
142	CDP-choline	7734359	295182	11143261	8472945	267766	286309

143	2-Methylbutyl beta-D-glucopyranoside	5574779	19320428	9828638	10949777	10634530	16346966
144	Ascorbic acid	6278354	1658549	1960986	4676772	40071344	13817631
145	2'-O-Methyladenosine	19008240	6960122	12553744	12163875	20072382	227197179
146	SKK	2468058	171178467	799775	1151804	2751377	1719280
147	FAHFA (14:0/14:0)	2682355	1167366	6840182	11854764	6352484	11421054
148	6α-Naltrexol	144299537	5903354	32797970	74337752	1377345	3694496
149	PE (2:0/18:0)	838555	642999221	2408230	398309	124336213	8583200
150	2,6-dimethoxy-N-(1-methyl-1H-pyrazol-5-yl)benzamide	3845392	37455587	1434175	1218400	2381051	1722307
151	R-Cathinone	3877990	15991666	7178000	56264514	15621089	56543234
152	LPC 18:1	1888228	644940711	6436927	960880	72085087	4686881
153	L-5-Hydroxytryptophan	592577761	617888632	372463578	345461106	776509589	2103237564
154	octadec-9-ynoic acid	4732264	52064463	4456938	3522272	190657184	201866522
155	o-Cresol	27733882	14056621	28671026	49916980	145507707	160118261
156	PC (14:1e/2:0)	1260915	162368097	31111959	2118180	48583346	22986515
157	Taurocholic acid	384243	34651513	532880	262303	6384442	10452256
158	N-Methylisoleucine	8439746	2465824696	5594614	7308543	1146127831	1060342689
159	Piceatannol	180776827	594258	154905003	148732729	20338250	14558911
160	D-Xylonic Acid Lithium Salt	1640805	7645302	1831770	3318074	128862165	198223696
161	NNK	21025004	14146242	46063164	17147964	34134171	5701735
162	D-(-)-Ribose	170817909	31256840	103581882	131784921	46829647	72947236
163	FPK	7002453	175703591	6457183	9661685	4184161	2474665
164	ethyl 2-cyano-3-[(2-oxoazepan-3-yl)amino]acrylate	23600325	159085713	21534988	19146347	179440140	132446068
165	EKK	5636294	170754962	5033982	2177578	2899855	2872661
166	Cucurbitacin I 2-O-β-D-glucopyranoside	6584721	29286949	9456516	11640612	4214523	880819

167	OxPE (18:1-18:1+3O)	287604	638323661	1105985	801643	186436344	2152049
168	Adenosine 5'-monophosphate	15330731	1611736832	106325192	14908415	227982653	46526560
169	5-Hydroxytryptophan	158850898	74042792	167957257	192816243	82035428	166114415
170	3-dehydrocholic acid	1893734	6485312	1645165	1550250	5110176	191550177
171	(±)9(10)-DiHOME	13754768	137018145	11728350	5570179	297020574	5833000438
172	SM (d15:3/18:2)	333005	3985676	379776	372691	188070691	22379214
173	LPK	12108337	167758285	7096032	6987380	6389945	4008100
174	2,5-Dimethylphenol	1879439	1461442	1678079	10578631	5794529	8487678
175	N-acetyl-glucosamine	9176135	2472775	2537797	9004633	3645148	8471185
176	Adrenic acid	1623399	510380683	1234147	2049242	181229556	569811872
177	11(Z),14(Z),17(Z)-Eicosatrienoic acid	10822958	177405663	1637535	1380664	65114162	122221603
178	PC (20:4e/2:0)	659295	172088729	1790624	701738	43088924	5594168
179	cis-Aconitate	7972039	2151908	10977297	5161811	29290511	15734313
180	Fluprostenol serinol amide	24331166	2093548	47470187	16093230	12231061	32560112
181	Ecgonine	7922906	164881390	8656445	9650360	138171567	73941591
182	4-(Diethylamino)salicylaldehyde	39951197	4960305	11748575	14263329	24832136	28302080
183	Ritalinic acid	1669648	2035042	1742129	51725775	3952729	3763008
184	Retrorsine	33016154	8176817	44998450	43468070	15062896	11648297
185	Pseudouridine	9764888	5639642	2904614	1674048	4624997	24335875
186	6,7,8-trimethoxy-2-(2-phenoxy-3-pyridyl)-4H-3,1-benzoxazin-4-one	648756	14472138	1080590	6823838	182676122	94437444
187	(±)11(12)-DiHET	10423949	8926992	4590576	2361246	1829610	600322
188	D-Saccharic acid	47337222	17468051	168225353	11385277	488628540	274466495
189	14,15-Leukotriene E4	162422134	496820	97529996	1020671	957852	1063368
190	5α-Dihydrotestosterone glucuronide	174418275	16677593	16921410	56469728	8373070	1167835
191	N-acetyl-glutamate	30971715	36982145	28790599	19163487	31040904	41138526

192	Methyl indole-3-acetate	10056248	1572342	4834022	4680792	3320820	3202610
193	1,5-Anhydro-D-glucitol	9847213	24360474	3383457	3477259	7542355	13584203
194	3-[4-(tert-butyl)anilino]-2-(3-thienylcarbonyl)acrylonitrile	1227460	16646593	1066143	641189	17670325	39898310
195	Cortisol	38031321	4132799	47023729	1063459	4639756	3224471
196	Sorbitan monopalmitate	9162175	2662630	1280057	847212	16050554	7436664
197	1-Methylhistamine	746534	703940	9866827	10298563	608346	669198
198	gamma-Glutamylmethionine	11063058	136382916	9120087	20248079	138217139	190757364
199	Glycyl-L-leucine	169322414	74458620	111949416	64239705	21345467	36025267
200	Ip7G	20297003	9464982	38036194	48248747	4641745	26356254
201	Estriol	5502949	23127613	6947250	7543626	24838993	203093209
202	LPC 22:4	389551	30082694	273549	227074	4040764	3173416
203	LPC 22:1	123410	34489312	159591	149851	19033212	1551789
204	PC (10:0/10:0)	538263	158645156	815193	633994	11587728	6145766
205	Kanamycin	31252471	18557985	45119849	5759625	24605924	1820865
206	MGDG (3:0/18:0)	119309	34490005	175482	136370	14021849	784143
207	4-oxododecanedioic acid	1913676	32212327	2467136	1135865	29504849	194743447
208	4-(3-Hydroxybutyl)phenyl β-D-glucopyranoside	8782641	457048	1744021	10701547	697380	4804399
209	ERH	1781124	159266355	2148892	1828611	2603624	2222980
210	IRH	38269244	5542982	9134171	16233266	9170951	16137235
211	FNH	20288393	3165482	42328764	46207558	16544717	19451313
212	Ala-Leu	37515436	12784088	23773770	20863664	16312762	8600916
213	L-(-)-alpha-Amino-epsilon-Caprolactam	33606823	39631559	38336289	8365962	31100760	26664179
214	OxPI (18:1-18:1+3O)	807228	31524483	540801	436179	5140107	4638995
215	VNH	19897355	5670488	44264227	3421400	27832110	48140550
216	Leucylproline	151710674	74313153	63460629	85728556	124236804	142674815

217	2-[5-(2-hydroxypropyl)oxolan-2-yl]propanoic acid	5658700	48341978	7064743	8093572	71214810	192067663
218	OxPE (18:0-18:1+3O)	338428	582467589	894747	679301	391982998	2035485
219	ethyl 3-cyano-6-methyl-2-(phenylthio)isonicotinate	802721	1115094	658767	683767	1005871	33286298
220	2-Arachidonyl Glycerol ether	1417704	120268023	1626578	1556922	162726688	2862532752
221	HET0016	36980066	3267332	11316635	15064978	8531124	6353294
222	LPC 16:1	482318	573461215	1585783	231084	31190367	2245410
223	LPC 10:0	178621939	5076962	61032974	671823376	21520492	16050558
224	OxPC (18:0-18:1+3O)	341992	572933740	710764	512686	335129760	929065
225	Adenosine 3'5'-cyclic monophosphate	9393204	2292021707	252349441	17385341	1635946245	68738084
226	Capric acid	7055470	15599056	8603985	3064779	126683211	188847529
227	Cytidine-3(2)-Monophisphoric acid	783169	18639010	1947636	1158753	32847629	2256294
228	13(S)-HOTrE	4672868	5815045	1486796	3735601	10183979	30001597
229	L-Alanyl-L-leucine	31731719	18090994	19927563	21954883	45880240	42377881
230	3-methyl-5-oxo-5-(4-toluidino)pentanoic acid	189285565	8926250	704408782	1470642250	30005556	21037226
231	LPE 20:5	757318	154913563	1392119	942695	8721484	6966540
232	1-[(3,5-dimethylisoxazol-4-yl)sulfonyl]piperidine	24007529	117472215	39068134	43182917	38744262	139057756
233	PAF C-16	1802263	554213876	3920579	2297300	296349651	11847690
234	LPC 19:0	128750	32116631	119982	246733	23465217	2379965
235	APH	1557623	142398465	2084658	1618959	75932083	1822933
236	Milbemycin A4 oxime	3374002	11159888	606475783	64114945	80410454	3379512
237	LPC 20:0	122116	31525901	116596	129209	31389564	1452740
238	LPS 18:1	1241841	4925239786	3355129	927570	557272410	46443578
239	Phenmetrazine	33207019	3784887	4140198	14636330	10601202	9740668
240	PC (4:0/4:0)	34752383	9091075	10219892	27785205	23168389	102245119
241	cholesteryl sulfate	593181	480412	537428	314888	688175	33339380

242	OxPC (16:0-18:0+1O(1Cyc))	454352	478245097	1404967	2035527	306639081	1719761
243	Citric acid	7602628	13728128	90342192	14790918	487870105	188068145
244	Erucic acid	6073066	30524591	3975072	4139142	155816478	474523388
245	Acetylcholine	67480441	39766008	430675355	664585058	74902255	80273428
246	Flavin adenine dinucleotide (FAD)	1357516	20433193	1719296	1406954	6966317	962500
247	3,4-dihydro-2H,6H-[1,3]thiazino[2,3-b]quinazolin-6-one	2690784	15777929	2239608	7472595	78739870	136707060
248	LPA 17:0	261249	29965922	405588	215070	4848401	891079
249	OxPC (16:0-20:3+2O(1Cyc))	190009	28451805	176429	169580	11401139	679308
250	WKK	23374991	7823373	40783431	26396842	11270313	8416555
251	Phosphopyruvic acid	750541	3847965	2951447	7677504	9147999	30270342
252	Vincristine	3585680	2718236	4900079	37334502	2464850	2122378
253	LPA 18:3	178784	29060514	192501	172426	1439521	1161732
254	LPS 20:3	118639	29947275	116070	127109	3084835	789102
255	Tetranor-12(S)-HETE	929424	8071818	2929240	1775119	21031801	185280358
256	Benzathine	15067706	2170355	40384896	14890833	2875895	2537748
257	D-(+)-Tryptophan	11697226667	2020803065	141926911	133196305	78492572	49887647
258	Nonanoic acid	10631625	23057735	69228470	144502607	42343599	47263260
259	4'-(Imidazol-1-yl)acetophenone	34647426	11609442	21901518	30156438	22215851	27647499
260	LPS 18:0	652847	467528084	986553	994080	550406648	36968119
261	Camptothecin	23520159	67605735	29963831	37141400	1079667787	2140602830
262	Corticosterone	4216767	48216116	3646675	8394617	94986825	157005333
263	PE (8:0/16:0)	187381	29750319	178911	186917	21298275	1292665
264	Sulfoacetic acid	4755345	27976939	4344397	4450086	15872354	18260968
265	PC (20:3/20:4)	428636	146353009	917508	448834	44165983	3617206
266	Hypoxanthine	1278018557	2921770298	1329022930	14239205	248818758	208459122

267	PLH	3479206784	35900656	15782360	17240400	17762844	27208516
268	L-Cysteic acid	1391720	22817110	2201359	1838012	1309254	4275459
269	N-[(4-hydroxy-3-methoxyphenyl)methyl]-8-methylnonanamide	6089564	79092607	17672023	20378039	97571044	163251748
270	L-Acetylcarnitine	2201263	25690424	1887689	1424806	14639714	11300894
271	Rutamarin	17704070	4803712	112878041	635152070	5498774	174519103
272	Phosphocholine	5348957	1779656062	16681477	8774461	184494559	33218985
273	RNK	1651815	129698194	1595662	1436609	2266366	1077007
274	S-Lactoyglutathione	21367250	2925295	37930227	25721414	2713563	4920770
275	N-Acetyl-D-galactosamine 4-sulfate	1378518	28872661	338162	1359741	2083983	3721215
276	(5E)-7-methylidene-10-oxo-4-(propan-2-yl)undec-5-enoic acid	3660126	4523160	11456694	7005664	156700406	4845305
277	L-Alanyl-L-Lysine	12926839	117324197	9739505	15162263	20858684	15633487
278	LPS 22:4	126794	26762515	123077	123022	4894817	890337
279	Arachidic acid	118083269	45811818	27681165	24006594	188147745	406966008
280	Creatinine	2399663868	904098630	3370872519	3915992043	787863797	1764337974
281	8-[(4-phenethyl-1,4-diazepan-1-yl)sulfonyl]quinoline	2630532	9003369	2571583	4822803	79312717	172047287
282	20-Carboxy-Leukotriene B4	2543764	122849336	13662758	7530324	5810189	3216892
283	DL-o-Tyrosine	19382307	36590799	18882990	41253548	36745903	39601984
284	Cynaropicrin	764616	25298727	772672	650654	13265079	10670067
285	C-8 Ceramide-1-phosphate	698450	131741960	1370702	781334	66460355	21546479
286	PC (16:1/18:5)	1010050	117869122	1023382	1034646	107781070	34741006
287	Cer-ADS (d16:0/18:1)	108636	1859113	105449	101850	4664440	20489576
288	Guanine	146081420	4506520907	101807425	10034558	91237027	34933658
289	HPH	1487920	108272608	2004758	1894265	28714141	29286628

290	Hexadecanedioic acid	2530198	2007588	2968492	3371973	4722002	509916460
291	Deoxycholic acid	33609618	1249061349	23457562	16393884	4804171447	289657751
292	Cer-AS (d18:1/16:0)	233372	3024884	208216	233476	6077840	19032544
293	PE (2:0/18:1)	306634	445729096	286897	284212	19166219	3847681
294	LPE 16:1	1367856	1967278467	28197035	2169738	127061998	22717176
295	Naringenin	7220790	699441	5009506	4680124	976180	672673
296	D-Fructose 6-Phosphate-Disodium Salt	1556375	729100	6656107	7467661	5386840	1039565
297	Serine	615264	1418911	1551184	422445	3486070	27078716
298	OxPC (16:0-20:4+1O(1Cyc))	254811	22887002	246548	240439	8005651	807257
299	OxPE (18:1-18:3+1O)	105055	25316333	100463	97261	8435096	323002
300	5-Methyldeoxycytidine	117560326	4671357	7928650	11618824	6759379	7294631
301	Suberic acid	811522	2665639	739389	710088	4016417	28713620
302	α-Methyltryptamine	26961298	3850511	28263577	37602664	9128491	20073611
303	OxPE (16:0-18:3+1O)	174186	23814242	166554	160787	12591373	1137359
304	OxPI (18:0-20:3+4O(1Cyc))	129955	25916395	123806	117666	16576538	1494917
305	Tetradecanedioic acid	5835528	12372592	6343812	13050490	21192218	469111144
306	PC (13:0/13:0)	1262641	130525051	1109592	918211	93510409	3644639
307	Phloretin	6647331	555513	6416802	5102424	617434	566990
308	Catechin	17943615	6584017	30131185	1653135	10458144	5872865
309	Glycolithocholic acid	616803	26637968	825525	554829	112014699	27839464
310	Oleoyl ethanolamide	4140482	16708341	2034818	1804325	65093937	150370763
311	15-keto Prostaglandin E1	431056	3484556	421992	372142	27284773	16524516
312	LPA 22:1	206135	25194458	1222648	326379	17091096	7777278
313	5'-S-Methyl-5'-thioadenosine	3197081	3261685	6111479	5072804	12157998	7995067
314	Elaidic acid	538100113	39335115779	562427395	359197585	24477341150	29162271484
315	LPE 15:0	653910	124977416	1663010	1212614	37992982	3215675

316	N1-{4-[2-cyano-2-(4-nitrophenyl)vinyl]phenyl}acetamide	15314756	3160475	21962363	36597045	6438554	17792082
317	3,3,5-trimethyl-3H,11H-pyrano[3,2-a]carbazole	26972254	5935766	8189753	11725054	26682040	33982957
318	Heptadecanoic Acid	6826478	71991823	5783088	6472783	43076888	135396336
319	Cuminaldehyde	9883558	65740493	21403060	28148431	94159395	137123415
320	Theobromine	21484390	174034596	514319847	2867387417	13802022853	16739198884
321	PC (16:0e/6:0)	1315872	98560951	1292841	1324314	128493162	9564787
322	PC (18:5/22:6)	546365	110648811	657950	641174	18406522	4352387
323	ILK	19813935	1819883716	7559615	6701607	31350206	21838101
324	N-{6-[4-(tert-butyl)phenoxy]-3-pyridinyl}-4-(trifluoromethyl)benzamide	95745054	1185689	129153606	59260661	8246483	5928171
325	PC (20:5/20:5)	191466	113193224	212218	230994	50472947	1535318
326	Stearoyl ethanolamide	6204288	24273758	3520624	2921714	27194648	129896597
327	DMH	3883607	93608457	4426850	5144431	3590230	4352637
328	7-Aminonimetazepam	317051823	4421426	507191194	469100348	148164052	23912840
329	LPA 20:2	207503	22404090	188399	96057	1135237	1111318
330	Esculin	496621	2543270	348716	311230	8868015	20780498
331	3-(3,4-Dihydroxyphenyl)-2-Methylalanine	6715037	1236362	953898	901443	1181690	1694252
332	LPA 20:0	237387	3027214	209861	186067	10533950	25738883
333	alpha-Ketoglutaric acid	15703432	14942505	117346779	15129704	272723630	128270567
334	PE (6:0/18:1)	296455	425337978	351694	285847	138056191	27744782
335	Glycylproline	24738355	13214697	5825969	6176134	12762350	14484471
336	Phenylpyruvic Acid	7234412	13547695	9557388	9259408	17482999	125271346
337	Inosine	6458042	3033935	33029754	2372370	18573410	5173122
338	PC (7:0/16:0)	113353	23192676	128947	103522	8690415	351904

339	6,7,8-trimethoxy-2-[3-(trifluoromethyl)phenyl]-4H-3,1-benzoxazin-4-one	8938906	2731379	31317445	783872	2739893	2903591
340	FAHFA (2:0/22:1)	316569	16834315	350653	352137	8203113	21432500
341	D-(-)-Lyxose	33944966	20729594	31542833	11995471	142134035	327147424
342	PPK	216549903	19062864	428712840	28065359	90635493	6451690
343	CDP	6316222	5154059	6360976	3267869	12035294	10352724
344	FMH	59349027	1496704	474989776	4697402	49361540	78199562
345	NAT13-331713_POS	6289549	6275976	7301367	7189016	4822606	132734283
346	L-(-)-Methionine	386833859	122544347	13842420	22013184	26053170	15677292
347	Hexadecanamide	212789750	985917548	256659040	202883889	1776911545	1978629024
348	Glu-Val-Phe	78542388	3417872	67961907	124892905	22938443	10588516
349	ENK	350595104	4442176	17173734	45425089	11966383	5557344
350	ACar 20:0	414658	52761353	412516	525907	128106950	32250639
351	PC (19:2/18:3)	629476	116172618	1246689	931172	123151146	5173167
352	Propionyl-L-carnitine	2771462	7442933	2977559	34574269	6225741	12113368
353	SMK	3095336	4074386	31055239	17292941	5369656	5449280
354	WQH	18095940	471416	8006609	1903378	578123	303306
355	AKB48 N-(4-fluorobenzyl) analog	2190195	100320771	2111341	4602943	24125871	2114576
356	PC (2:0/13:1)	3132323	96463734	2109747	1719455	42214192	109037236
357	PLK	1885953	12685898	10247094	34305640	1578034	1112367
358	LPI 15:0	141913	21881235	152801	150716	1156951	945649
359	PE (2:0/16:3)	1633728	101036671	1076605	872429	39478098	54276065
360	1-[(5-methyl-3-phenyl-4-isoxazolyl)methyl]piperidine	16324869	17502520	30998483	17828414	9618616	6150555
361	Cortisone	11605647	6147498	27852368	17970051	29472657	4845538

362	4-[1-(dimethylamino)ethylidene]-2-phenyl-1,3-oxazol-5(4H)-one	29861340	1711062672	47406232	55032569	106335781	85253372
363	Kanosamine	164984850	32408248	123925959	4929970	35506207	2011300645
364	AKK	971279	102563860	732075	745337	2348060	2753925
365	Lipoic acid	5669113	11082269	34198829	25325425	185311399	449385670
366	OxPI (16:0-18:2+1O)	110144	20484040	102868	191357	4256006	494881
367	PC (13:0/13:1)	545316	110978149	738599	671351	47795601	2323034
368	D-Raffinose	20589657	1695317627	38478775	44412833	79853237	59094329
369	PC (18:2/22:5)	513308	20869186	366724	360354	6653183	4115277
370	Tyrosylalanine	119322064	2561143	28151228	52777827	4769654	5300009
371	DL-Dipalmitoylphosphatidylcholine	2962346	8090849	3302315	29569800	13465842	7418030
372	Docosahexaenoyl Ethanolamide	1788682	4961130	2270908	1576467	3632812	135588606
373	LPI 18:1	246525	3839284097	758362	192780	72224380	5166345
374	4-methoxyphenyl 3,5-dimethyl-1-phenyl-1H-pyrazole-4-carboxylate	25636922	3215847	10898206	21638050	5839083	12999363
375	3-hydroxy-3-methylpentanedioic acid	13588617	99213742	16344063	14420488	113582834	93620591
376	N,N-Diethyldodecanamide	30514182	1574135375	29627747	45170469	820592676	620703092
377	N'-{6-[(5-chloro-3-pyridyl)oxy]-3-pyridyl}-N,N-dimethyliminoformamide	23870109	8953114	9956739	7927398	15424277	13474848
378	Ethylmalonic acid	3236152	10221968	3778658	8516395	120635349	311736284
379	LPS 16:1	139064	20920528	260564	156103	1169481	1833788
380	ethyl 2-{[(1-methyl-3-propyl-1H-pyrazol-4-yl)carbonyl]amino}acetate	25660616	9130592	13549471	26466101	16587839	17156574
381	1-{4-[2-nitro-4-(trifluoromethyl)phenyl]piperazino}ethan-1-one	1469448	4286688	1809998	2563075	59123646	113341438
382	Hydrocinnamic acid	423223921	147726809	83668186485	96273546546	539053165	602065517

383	Styrene	90619367448	2476355704	94409142022	104753348552	2161764624	3018014016
384	(±)9-HpODE	2445400	3721856777	3176417	3847992	421383486	517606380
385	2-[({4-[3,5-di(tert-butyl)-1H-pyrazol-1-yl]phenyl}imino)methyl]phenol	3628856	21864671	5195183	4236905	21592553	133846436
386	Tanespimycin	24730230	21396585	20648048	19153121	42930330	3017840
387	Thromoboxane B1	880808	3367594	1236381	870857	3351444	22476035
388	LPC 4:0	4963660	19707858	3458428	5009016	26403016	103986589
389	Minocycline	12012818	4435972	28969633	18854297	5299654	7479193
390	Creatine	2245467279	663091357	3158335890	3701530496	986560531	1088310166
391	Cholic acid	1339177	1502013	1171765	691394	9724614	446269245
392	PE (4:0/4:0)	14595921	94264787	12054091	13989525	52559851	133587779
393	(6E,10E)-3,7,11,15-tetramethylhexadeca-1,6,10,14-tetraene-3,5,9-triol	1633507	8758238	1688565	1126470	7623513	123064558
394	Guanosine monophosphate (GMP)	8102460	381191763	61947468	2278618	249395035	39997406
395	PC (11:0/11:0)	643821	102247181	709823	814915	38676715	4791877
396	D-Glucosamine 6-phosphate	307728	19173365	648501	269595	2817821	2809524
397	1-Acetylimidazole	5892899	9597725	4990142	2116014	44421735	105641202
398	N1-(2-oxoazepan-3-yl)-N2-(3-pyridylmethyl)ethanediamide	20407456	1562664	18085826	26785065	2461955	3756027
399	RMH	14855749	2066715	27756169	12540975	8943575	1907826
400	2-(2-Aminoethyl)pyridine	23482009	6326143	9532003	11845909	20128464	31507083
401	N-Oleoyl Glycine	1216339	100139129	749076	664754	23264634	40362330
402	trans-4-Phenyl-4-piperidinocyclohexanol	4035162	71203602	291507339	464120096	28326468	42083076
403	5-Methyltetrahydrofolic acid	1045899	960145	1367179	1389018	870228	20506101
404	LPC 20:4	1037917	386416085	2339545	560830	29460407	5964720
405	PNK	14720619	7467223	4811740	29546385	5425281	5958841

406	Guanosine-3',5'-cyclic monophosphate	6945219	371958984	60762183	966283	248825910	35180792
407	Caffeic acid	6155099	6976394	19907704	28960163	114768889	1991800693
408	LPE 6:0	6713542	39770321	9491429	8109253	32432076	81742168
409	Hendecanoic acid	111687163	24363582	99286756	77140674	107543937	260024214
410	(2β,3β,9ξ,17ξ,22R)-2,3,14,20,22-Pentahydroxyergost-7-en-6-one	517440	5899649	524435	627688	16396225	107046414
411	Androsterone	20904715	4094715	26458283	29881506	15277718	27929608
412	PC (16:0/17:1)	2534485	16464420	17273686	27130442	17673610	12860368
413	4-Hydroxyretinoic Acid	459692	1814022	447577	399606	3675646	17389594
414	(R)-(-)-Epinephrine	367615380	335945929	288950910	247436260	459630846	605050425
415	(+/-)5(6)-EET Ethanolamide	22846645	6224665	560338	563825	4492621	11438304
416	thiamine phosphate	4982488	4762346	727370	1603102	1705523	1764953
417	11-dehydro Thromboxane B2	869600	3337623	677397	657846	5206964	17904156
418	VPH	199973650	103109257	199793453	467058673	50818420	4867181
419	3,3',5-Triiodo-L-Thyronine	368249670	12440956	14707996	13697526	408124094	5691009
420	2-(acetylamino)-3-[4-(acetylamino)phenyl]acrylic acid	14375758	11284603	17328361	29000372	37131263	76062445
421	DL-4-Hydroxyphenyllactic acid	165840027	42359070	233794139	293514133	814658699	3035962901
422	YNK	7318371	2759489	1241466	29824522	1601915	3101679
423	Calcitriol	50137008	53584792	41546304	30577629	401503319	281590280
424	Hydroquinone	2898903	2981460	759275	1911423	6064011	17054431
425	D-Fructose 1-phosphate	13746637	26942092	29363783	7169766	123033613	402840288
426	FAHFA (2:0/18:0)	2296150	1823482	1502431	5054938	5754325	2538083
427	MMK	22930216	4885461	22845072	22173019	12369335	18948327
428	5-Methyl-dl-tryptophan	7882146	14785499	19099546	26410431	15467070	18281021
429	Glycochenodeoxycholic Acid (sodium salt)	102473455	1902516	28255621	15466988	5304566	791794

430	2'-Deoxyadenosine	2654005	9295181	1458167	1423798	3218932	17579960
431	L-beta-Imidazolelactic acid	3745406	3698500	23202155	1700984	130848096	329744747
432	OxPC (16:0-18:3+1O)	301915	349147290	521005	268548	125477141	1368726
433	N-Caffeoyl Putrescine	371529238	7480710	360780934	116484905	47437030	1812885
434	LPG 19:1	515830	6959437	3662918	586852	18107848	2959844
435	Palmitoleic acid	2583821	82768194	3270382	5794658	57291642	78560710
436	2,4-diaziran-1-yl-6-(1-phenyl-1H-pyrrol-2-yl)-1,3,5-triazine	14946306	4472275	9110398	26911740	8628890	18184434
437	N1-cyclohexyl-4-(4-fluorobenzyl)-1,4-diazepane-1-carbothioamide	1254600	16672497	902949	811643	1105578	756709
438	8,12-iso-iPF2α-VI	13113064	305054441	9655911	8216855	200706341	341983412
439	CUMYL-PICA N-pentanoic acid metabolite	3596056	1559758	3612421	5304329	496845	828260
440	9-Oxo-ODE	511160462	2018131529	611147470	405917601	3401365638	15403613574
441	LPG 15:0	122876	15969980	3761948	927655	1211150	785659
442	PC (22:6e/22:6)	641274	85360729	651562	658733	48599395	4172267
443	1-(1,8-dihydroxy-3,6-dimethyl-2-naphthyl)ethan-1-one	99111823	3288791	88133935	94835758	56023255	28756057
444	1-Methyladenosine	2779287	3185687	6831635	27044994	4177100	6578339
445	Testosterone undecanoate	793672	5024569	3463686	28782641	5288970	11487530
446	Nicotinic acid	103466189	50185044	86657383	43860139	50428140	43305037
447	Methylmalonyl-coenzyme A	512403	13810492	464617	537534	3448714	262097
448	OxPI (16:0-18:1+3O)	450952	339064384	443774	260794	61709737	3031686
449	6 β-Hydroxycortisol	1070323	20851462	847653	26503457	4189952	3776591
450	farensyl diphosphate	19881912	2890171	23900620	5589444	15226421	4841173
451	PC (16:3/16:3)	114981	14334895	132904	100887	7332974	741573
452	1-methyl-3-phenyl-1H-pyrazol-5-amine	2054993	86692419	2880246	2358080	4842739	3301968

453	Chrysin	2398054	10469726	13641602	1152863	9317709	7511742
454	1-(4-Methoxyphenyl)-2-propanone	2944375	11272158	23526863	23433261	13651903	30030816
455	Flavin mononucleotide	721984	431453	2187807	4149042	382071	835905
456	Hostanox O3	638287	1107343	1471831	1849635	18015565	15235020
457	PC (17:0/18:2)	1633654	8481867	8459498	24293454	10766186	7687698
458	16α-Hydroxydehydroepiandrosterone	54691148	1088300013	40358773	83511466	1609247965	799908706
459	1-(3,4-dimethylphenyl)-3-piperidinopyrrolidine-2,5-dione	21165424	5614138	5957078	9800930	6644032	12941822
460	LPC 14:0	191803	354616896	596178	154493	36833088	1264309
461	5-Hydroxyindole-3-acetic acid	50295595	74223856	60380955	394340223	89497511	106903579
462	Stearic acid	2350552511	2833434163	1706413015	1622346908	18522587941	31708302692
463	PE (18:2/18:2)	756667	83232673	1177321	1282036	35489789	3118751
464	RNH	20779008	15213337	19189561	16784802	3532892	2036004
465	gamma-Nonanolactone	2657455	34179181	3492063	1606087	43359976	100513008
466	N1,N1-dimethyl-4-fluoro-2-nitro-5-tetrahydro-1H-pyrrol-1-ylaniline	2713284	16151244	2663095	26844387	8634516	11458230
467	Pyroglutamic acid	220216353	4537819431	2736536250	242395336	10541773570	1727882619
468	Hippuric acid	867439	3190019	751728	5689011	349276218	233481539
469	Lidocaine N-oxide	19900413	3102366	17782903	24921267	6481991	6365451
470	Aplaviroc hydrochloride	2449382	85034799	2179156	1358315	82815258	95984585
471	PC (19:2/20:5)	351177	47210300	371887	451969	73662349	2882674
472	1-Methylguanine	117972440	43037895	155083763	8092519	72720535	1712529529
473	16-Hydroxyhexadecanoic acid	212344308	3468234628	309320082	179340992	1858970137	1438471591
474	OxPC (16:0-20:3+4O(1Cyc))	127996	13894396	122028	124779	6484905	614636
475	1-(2,4-dihydroxyphenyl)-2-(3,5-dimethoxyphenyl)propan-1-one	2083962	14646145	1846623	1455284	7738870	16736200

476	5,7-dimethyl-2-phenylpyrazolo[1,5-a]pyrimidine	2198194	2134299	2852225	24184422	3750342	2176520
477	Glycocholic acid	1964199	1937426	1169995	206607	15872451	3673177
478	PC (12:0/12:0)	1820358	1410049533	3751676	2505369	724712178	5949683
479	N-Acetyl-L-methionine	829908542	11926010	534150738	381584330	4433941	7358251
480	(3β,9ξ)-3,14-Dihydroxycarda-5,20(22)-dienolide	498151	1958768	446855	504283	854961	14163017
481	Prostaglandin E3	95776027	622996	52406873	23090856	7384339	887636
482	PC (6:0/16:0)	178417	12585345	181347	172329	4377439	691364
483	PC (2:0/9:0)	595821	3510023	523659	314934	10857964	15754697
484	Soyasaponin I	96445576	2353175	67525364	223551	7635409	6216291
485	Dl-3-Hydroxy-kynurenine	14729872	15925679	20825427	21266090	19598396	29450420
486	Cholecalciferol	3013022	616570424	3976092	4325917	799553492	1488413443
487	6-Methylquinoline	12931952573	398270179	24319822546	24849592216	116391474	437386437
488	dAMP	3315484	14112103	3494447	1242013	6961546	2018549
489	Coenzyme Q2	2611400	16564944	2108369	2766738	37590822	99854663
490	Octadecanamine	17453037	6356550	13778384	23042886	11292131	10683502
491	Ethyl myristate	500725913	2225162049	314562617	267353405	3494127230	3277638819
492	4-oxo-4-(5,6,7,8-tetrahydronaphthalen-1-ylamino)but-2-enoic acid	17858761	83026337	19695327	14819791	35036008	49622763
493	QPK	17903819	4500212	4692245	4803259	3207703	3815935
494	Thromboxane B1	3696607	472024	2036211	415102	936976	1544594
495	PC (8:0/18:0)	104252	13148232	100916	102126	7905499	329660
496	N-[1-(4-methoxy-2-oxo-2H-pyran-6-yl)-2-methylbutyl]acetamide	18700034	3010864	8817323	9359160	6054585	9689265
497	16-Heptadecyne-1,2,4-triol	5842313	79919713	12137850	25369390	77168253	65574762
498	QNK	18252789	2958194	21323075	2463701	14224298	2167904
499	OxPC (16:0-18:2+2O)	441027	318181480	675495	753594	285909888	1038313

500	MQH	2087598	12182270	1722630	23482407	7374074	1927505
501	7α-Hydroxytestosterone	5837379	48149548	6901163	9355476	55859421	87005900
502	2-(2-hydroxy-3-methylbutanamido)-4-methylpentanoic acid	16956504	48527892	7602926	24955688	53742739	70342428
503	Isobutyryl carnitine	5798139	71185199	7283888	7010234	82732504	68583431
504	4-Ethylphenol	927885	530152	43533962	99364561	8134830	60321453
505	SM (d15:3/13:0)	783398	81301268	750487	792173	5841400	5334551
506	Thymidine	91920642	18821260	67879871	79246512	37236857	17068511
507	Estriol 17-sulfate	839481	464713	829194	813409	7735678	15171659
508	OxPI (18:0-18:1+3O)	115224	301037495	105192	99072	85298632	758208
509	YLH	11162100	71085227	7265955	7931745	16481024	21684301
510	(S)-N-α-benzoylarginine ethyl ester	13099221	5025957	16566986	21747114	15244508	41395383
511	PE (2:0/16:0)	113193	309000275	113574	107692	8490919	852324
512	Imidazolelactic acid	127423349	13781110	223253722	3310731	375593190	705830982
513	LPI 8:0	532929	13286429	481319	290240	6126420	1559837
514	Taurohyocholic acid sodium salt	396791	12034688	244407	164570	3833850	3932064
515	Psychosine	8079341	1345300972	3523507	2096389	459682482	145755017
516	5-Hydroxyindole	8596954	59393315	12904575	21564953	57245611	97503454
517	8-iso-15-keto Prostaglandin E2	164761	845734	175797	142415	14623217	8130622
518	Serotonin	12055514	13095226	144262028	381407573	126440060	137162820
519	VMH	3564097	74553832	6588780	3060431	6562536	3614043
520	5-heptyl-4-hydroxy-6H-pyrido[3,2,1-jk]carbazol-6-one	1721984	11625014	1696348	1654149	1012502	600336
521	Lipoxin B4	228049	674478	267045	213470	14309324	3152833
522	2-(1-{2-[(3-furylmethyl)amino]-2-oxoethyl}cyclohexyl)acetic acid	91783880	2220057	12215774	42385259	7884951	13019899

523	PC (18:1e/2:0)	1698654	1195704268	7320324	3608322	642442872	31008726
524	PC (11:0/13:1)	535635	73209259	583413	644414	52227189	4212095
525	AMK	17972785	11718774	15534684	5245102	11021768	4864470
526	2-Arachidonoyl glycerol	4201475	19131212	983574	639157	14016069	88915578
527	LPA 18:0	101742	12931661	194377	128600	5508356	592716
528	L-Saccharopine	3204019	1260348	3738294	672241	2121727	1771516
529	PC (18:5e/4:0)	611877	77150446	996014	648854	6092142	5566040
530	Actrarit	14721890	31336721	10493336	14925624	58920727	86379849
531	LPE 18:3	1149630	1178698014	2958677	994631	128376095	5738780
532	5-(2,5-dihydroxyhexyl)oxolan-2-one	1275807	9533516	960534	724364	26300791	87888167
533	Sorbitan monostearate	140870	3050817	261852	264328	5325315	9942263
534	Ascorbyl palmitate	501346	7522099	231836	176988	10697390	4632955
535	MGDG (2:0/18:0)	523114	12829043	498058	481010	6148293	2088278
536	α-Ergocryptine	5380238	70701019	5089768	15478635	11098223	25199762
537	13,14-dihydro-15-keto Prostaglandin E2	89276555	3176425	17073922	23334818	3402961	3877692
538	JWH 018 N-pentanoic acid metabolite	985110	2408584	975945	874666	5637439	13780007
539	OxPC (16:0-18:1+2O)	638501	300882738	1046801	680059	167712636	2088070
540	Fexofenadine	17278904	59675440	11203651	12411150	41327944	15091340
541	VLH	4648021	1199932809	5578366	7544969	49374854	38244248
542	Prostaglandin G2	225975	1132184	271114	107968	8588416	11989602
543	(+/-)11(12)-EET	344040	1445665	1086213	2276132	13552164	2178383
544	LPI 18:2	229792	3093186106	330497	182372	5319256	1714914
545	beta-Nicotinamide adenine dinucleotide phosphate	680470	72512067	718050	685805	1695937	2108358
546	Nonadecanoic acid	12878428	21857282	10334690	7673685	124851169	258143755
547	3-Hydroxyanthranilic acid	41627838	81790167	61226887	386993438	97298354	550935212

548	N-Butyl-2,2,6,6-tetramethylpiperidin-4-amine	1378036	69122450	1683876	925570	3112320	2287766
549	Nor-9-carboxy-δ9-THC	1874582	11273921	2393451	2087126	5323689	5178612
550	Valeric acid	746798582	102719191	3406173289	8670593303	262178537	726101776
551	Phenethylamine	18334278566	356196063	52827730	97796945	224602026	288475735
552	PC (10:0/11:0)	711243	70737650	722715	730669	14439552	8026931
553	1-[2-(2,5-dimethyl-1H-pyrrol-1-yl)-4-nitrophenyl]-1H-imidazole	2097686	2967493	2236493	2318338	5578988	88714861
554	PC (16:2e/6:0)	743688	73475191	844486	770598	47141293	14442934
555	6-methyl-5-nitroquinoline	3361255	8650096	3088437	3688324	6076555	6477793
556	TNK	16526251	30185668	17120993	19368561	9329720	7446974
557	LPS 18:2	171162	291473563	139984	127114	3862347	1000621
558	Paliperidone	11360156	2135405	20114299	13779911	3372831	4523108
559	CYM-5520	17043896	2989566	6059965	3592881	3093600	3158643
560	Gly-Tyr-Ala	16995370	8000717	7169572	10695279	8976604	23497317
561	Prostaglandin F1β	97531	294325891	92121	96566	27106074	893253
562	3-(2-methylpropyl)-octahydropyrrolo[1,2-a]pyrazine-1,4-dione	2419953	388572	5454712	22288069	405038	6909832
563	N-Acetyl-DL-tryptophan	54757063	1625208	76028870	93314348	4715087	6871717
564	Urocanic acid	67544249	21325505	72061552	7006990	25708399	17817114
565	4-fluoro-N-[4-(4-methylpiperazino)phenyl]benzenesulfonamide	1067017	11219733	1040714	631489	3127826	1022851
566	8-Iso prostaglandin A2	663670	4236816	598745	323125	2133107	12876874
567	2-(1H-indol-3-yl)acetic acid	14056500	68816382	13853397	16788194	42532376	63807921
568	17α-Hydroxypregnenolone	2560913	7733134	1137993	670464	4354324	2698833
569	4-(octyloxy)benzoic acid	3363828	8572786	620494	955971	3301354	2578150
570	N-α-L-Acetyl-arginine	13124012	3389871	13403609	21929167	17735610	22845944

571	LPE 24:1	87258	11011232	92149	84282	12314564	3093799
572	LPI 16:0	294030	3060829337	509320	251529	112277060	3324458
573	PE (6:0/16:0)	549036	291061385	367826	296667	67862231	3155210
574	N6-(2-furylmethyl)-9H-purin-6-amine	16119399	1090962957	21313645	30318110	785606677	1316885033
575	7-methoxy-1-methyl-3H,4H,9H-pyrido[3,4-b]indole	16152704	11817236	9771929	11641421	14052554	18757923
576	Octyl hydrogen phthalate	4568467	3628064	4962605	15064339	3328069	76839792
577	LPC 22:2	239516	9256148	244172	254681	5647169	1414116
578	PE (8:0/18:1)	183267	10982260	169971	175316	6742250	586913
579	N'2-(2-furylmethylidene)-3-aminopyrazine-2-carbohydrazide	2930195	66631780	3191631	3174259	36533160	85497297
580	QKK	1634890	62703627	966028	748921	4422506	1314878
581	ACar 18:1	1122273	68607535	912707	952260	14476844	6960769
582	PC (8:0/18:1)	126213	11356854	124500	121196	5580130	378023
583	ethyl 2-methyl-1,5-diphenyl-1H-pyrrole-3-carboxylate	16061841	11097109	4098450	5242986	9899879	7766051
584	D-Alanyl-D-alanine	26421558	8477220	51912548	79486049	14441589	43241729
585	(+/-)8(9)-EET	807448	13957928	1411344	1323973	49476274	74708213
586	(2E)-3-phenyl-N-(2-phenylethyl)prop-2-enamide	2602697	4440423	2652478	2578092	4014061	78508943
587	LPA 20:4	285145	10584059	815593	248165	2110040	1948938
588	Yessotoxin	156378731	13060395	270038718	355799411	266772370	10810526
589	LPC 18:3	198061	253452039	762900	186267	12123119	1818316
590	Oxoadipic Acid	9459154	2835163	43183056	55759764	102452619	50640414
591	PC (15:0/16:1)	731894	6698283	1273370	15205803	7206592	6228579
592	Lithocholic Acid	611937	17613133	1502691	320063	319392921	11149876

593	1-benzothiophen-2-yl[4-(2-pyridinyl)piperazino]methanone	2595016	5757427	1803509	3804547	2375210	79856002
594	6-O-Isobutyryl-alpha-D-glucopyranosyl alpha-D-glucopyranoside	8737025	3686875	17480134	5583178	62482046	3604137
595	L-Aspartic acid	34365342	10733152	74473434	40091220	36680169	15566789
596	3-hydroxyquinuclidine-3-carbonitrile hydrochloride	6626435	133112943	8232430	10245357	1058653979	218769806
597	N-benzyl-3-(4-chlorophenyl)-4,5-dihydro-5-isoxazolecarboxamide	1148012	534406	1216422	997245	654829	9754517
598	Cystine	28899709	4953035	25163972	90852347	7397950	11952538
599	CYM-5442	200847322	9554663	312292735	189429019	84925868	32827604
600	WNH	3088922	23261000	4043980	3171004	50642404	78661578
601	LMK	2069721	64865895	1394339	1380781	1590680	1248712
602	ethyl 3-cyano-2-hydroxy-6-phenylisonicotinate	9963149	1737825	77244141	1041191	20222247	3531100
603	LPE 17:1	2847926	1002786980	160739401	6005652	127782210	41530616
604	Andrographolide	2707391	24623688	2661391	3537797	23763150	79062855
605	SPH	2766069	57567079	6163857	5624074	29539164	2411539
606	(±)10(11)-EpDPA	141224	620856	2947686	2746516	946632	697639
607	Sucrose	165173804	2274914460	44429566	10946847	165377263	200797089
608	EPH	56463065	11076386	69611139	316058214	19161836	103361048
609	Kinetin	10628942	1137894760	8527563	2292459	254224260	1251661794
610	DL-Lysine	1283344682	139111988	2334102748	145694024	740049813	629052504
611	5-(tert-butyl)-2-methyl-N-(5-methyl-3-isoxazolyl)-3-furamide	63917291	1783372	64267410	84209630	15252110	6439838
612	dCMP	1870638	9436794	1179012	569643	1939474	1344814
613	L-Asparagine	11210712	32138723	7534112	11425977	36512392	58859258

614	GlcADG (12:0-16:0)	60534	7929399	58019	115821	6106658	963671
615	delta-Tocopherol	273385	32729439	276498	295938	43789078	73768024
616	SLH	1865614	3530097	9401058	18631673	2250581	1567485
617	Anandamide (AEA)	1884700	6157359	880522	930232	27357322	76664510
618	UR-144 N-(2-hydroxypentyl) metabolite	263096568	145602456	100788430	72230063	327392924	64403918
619	6,7-dihydro-5H-dibenzo[d,f][1,3]diazepin-6-one	260328185	46961037	2616254	4105573	4090085	4560054
620	6β-Naloxol	71699242	2166089	36067440	5553299	18908211	21601329
621	D-Arginine	2337120	9763861	1880004	533986	1567049	1231077
622	D-(+)-Maltose	64484474	905193581	24642701	17081599	173429369	181793541
623	WLH	3921804	61043315	5735826	5045054	8750894	7931160
624	LPE 18:1	11899787	21570580553	63906286	9916794	3157182752	107518149
625	Oleanolic acid	5590819	8618551	14658598	2386244	8380698	7675849
626	6α-Prostaglandin I1	973161	2196355	129820	107000	9958404	2751163
627	3-Methyl-1-phenyl-1H-pyrazol-5-amine	2749083	61036370	3792504	3787991	12178081	15313863
628	MN-18 N-(5-hydroxypentyl) metabolite	2305160	9011091	1488036	2702577	3121396	1836610
629	LPA 7:0	1384250	9244186	1097906	767125	3759636	6950759
630	PC (2:0/16:2)	5879337	9972035588	21917150	4728159	601451051	37526470
631	LPC 15:1	608662	52216831	747669	787563	15837333	5841901
632	PC (12:0/13:0)	680513	59644073	704258	880865	35737660	2755655
633	Glycerophospho-N-palmitoyl ethanolamine	21754806	9633790034	111017790	22843586	4369121664	164155423
634	LPE 14:1	194395	8132402	427127	260558	1411874	804613
635	DL-3,4-Dihydroxyphenyl glycol	874091	942845	728607	769880	1178002	9339920
636	NPK	13831560	17081279	5776455	7607404	8507307	2297602
637	PC (16:1e/2:0)	33308266	9496260962	180255190	16238416	464633258	133455651
638	7,8-Dihydrofolate	2033669	1090118	2012415	1981404	3598862	8512265
639	Tetrahydrocorticosterone	349971	56656690	401220	413711	27316204	3357862

640	16,16-Dimethyl prostaglandin A1	1468427	57319489	1423723	1263555	65525077	1579374
641	4-oxo-5-phenylpentanoic acid	791680	47820040	893091	940028	1830881	2391909
642	PC (22:6e/8:0)	219733	56220575	259164	239921	26220906	2706942
643	Guanosine	69963519	6508920	74839374	1394199	61755716	19417194
644	Hexanoic acid	13975629251	281271792	57758522019	57536853834	4102622598	5538540718
645	D-Isoleucine	76321501951	25598484312	15909771774	52433412658	7376711649	2167248553
646	Xanthine	5570341181	1207333107	4022029331	6088796	234197082	687395591
647	LPE 7:0	5119683	55041569	6282050	2643627	35337467	43441244
648	Stanozolol	174873546	3214065	272197457	40536140	41951448	44415260
649	PC (22:6e/13:1)	657751	58329320	665087	673249	37838206	6281563
650	Troxerutin	3640428	18068802	5084399	5885187	458287224	1201640632
651	PC (20:5e/11:0)	873596	27822667	884703	1044458	64146100	6197704
652	Artemisinin	11447832	1414035	6402892	11589808	919298	1010511
653	(5ξ,9ξ)-17-Hydroxykaur-15-en-19-oic acid	200965	5176138	181939	182069	3711333	7888089
654	4-Hexyloxyaniline	5485552	17053887	7251893	6491095	20496459	72096293
655	3,4,5-Trimethoxyphenyl 6-O-pentopyranosyl-β-D-glucopyranoside	1294645	1555188	5942589	14013393	1463797	1912340
656	IPK	1884603	53449520	2457031	2011435	7823974	1707267
657	1-(7-methoxy-2-oxo-2H-chromen-8-yl)-3-methyl-2-oxobutyl acetate	607438	4654239	1103749	1443395	35483400	59857048
658	LPC 16:2	736265	52401621	719056	810196	6804742	9459614
659	8-Bromoguanosine	211685	5054068	196343	866511	7706508	3969460
660	Nicotinamide adenine dinucleotide (NAD+)	2614707	2505793	11058081	1769893	6069925	3990009
661	6β-Naltrexol	70479456	2984470	271117639	4748765	35878463	6981797
662	LPE 16:0	737511	56979360	1036583	845169	11613498	2812591
663	Octopamine	1042078	620764	2000563	2361476	569122	496434

664	Cytidine 5'-monophosphate (hydrate)	2167109	10733279	15349654	11425924	4686070	5160832
665	ACar 20:1	448034	53210277	460504	363124	38670543	32803875
666	OxPE (16:0-18:2+1O)	234885	247679547	426720	248067	84092084	2118703
667	OxPG (18:1-18:3+1O)	63971	8027246	55073	53347	2665979	293427
668	2-morpholino-1-phenyl-1-ethanol	2765901	4430825	5251478	16181164	4115780	5470924
669	Uracil	155594103	114821204	394841681	557413547	189935245	2746102585
670	LPC 7:0	692072	6288154	717956	304076	6618279	8819368
671	2-(1-benzyl-1H-imidazol-5-yl)phenol	2152806	766806	195918	239450	930909	1255416
672	Flavanone	617927	812692	607529	574990	7876816	2140983
673	Propylparaben	471095	5887044	471428	607533	7393755	7900940
674	6-amino-1-(2-methylphenyl)-1,2,3,4-tetrahydropyrimidine-2,4-dione	6346426	997053024	6031027	4004156	220062353	343385503
675	XLR11 N-(4-hydroxypentyl) metabolite-d5	35874681	145134810	38016123	45650182	722088363	1233830480
676	N'-[6-(tert-butyl)thieno[3,2-d]pyrimidin-4-yl]-4-methylbenzohydrazide	5969987	978292	1308341	9534279	1178610	1053278
677	LPA 8:0	515802	7595894	376567	217921	982773	8118609
678	4-(Diethylamino)benzaldehyde	1746189	3155737	6890385	15709471	5637966	6395201
679	Stearamide	140488307	298745818	170758946	145103061	585269815	1001413876
680	PG (18:1/18:2)	59610	1118520	58132	56645	8091506	598304
681	3-Anthraniloylalanine	216815089	136135162	147183757	114828508	249825006	360495251
682	ϒ-L-Glutamyl-L-glutamic acid	12105994	2063608	13103158	2345617	1793807	2662623
683	LPE 21:1	100418	7177077	90451	87803	4202386	408848
684	15-OxoEDE	223108	629717	197076	165553	822672	7737946
685	(±)5(6)-DiHET	2100074	236521969	1769520	1801183	109676855	111797652
686	N-Acetylaspartic acid	56035069	19285179	63213984	69054268	169412595	173180806
687	3-(4-hydroxy-3-methoxyphenyl)propanoic acid	11423167	14744573	7659120	13668120	23864704	29663242

688	LNH	8608341	1991562	14438065	5546180	4015877	16039070
689	1-Methylguanidine	6910391	10353212	9121821	8803912	9846832	63346425
690	GPK	213583316	8779576	7488544	14869247	5694510	6194408
691	LPS 9:0	1368674	6321765	1508048	1146186	2397440	4496417
692	Dihydrokawain	997306	51331240	939965	837825	27415685	41059755
693	OxPC (16:0-18:1+3O)	1466953	2610936137	4443206	2510228	1142208819	3701569
694	2-oxopiperidine-3-carbohydrazide	229410794	8093746	159472074	139873107	46955182	44047491
695	YKK	10480735	3357880	12643516	15602146	11556696	2271178
696	2,3,4,9-Tetrahydro-1H-β-carboline-3-carboxylic acid	230807641	223218797	168193858	277057296	241302091	598425529
697	N-Acetyl-aspartic acid	10552075	18035517	11858571	13780815	32432510	21729711
698	LPE 20:4	6406186	8605796339	27238634	4683189	1558641191	42150016
699	13-Hpotre(R)	4033382	156710045	4522601	4542122	93492735	243676014
700	FPH	5542333	3384612	7063041	13868723	11772354	10101360
701	LPH	10892412	43567403	4763917	15014903	3005697	10842850
702	[3-(3,4-dichlorophenyl)-5-isoxazolyl]methanol	7524069	20514288	12001918	14675653	17523479	29511430
703	Salsolinol	48126634	5992555	67351546	74131562	15801292	17197143
704	5-Aminovaleric acid	7555199651	1042356099	15007371490	9242364440	1870833338	2890731617
705	Cystamine	5497519	48243441	6754690	10799479	49336927	55634003
706	6-O-(2-Methylbutanoyl)-α-D-glucopyranosyl α-D-glucopyranoside	143825766	5779000	152102705	198515898	8210406	6553324
707	Colchicine	4769395	55915415	6244035	4896132	463714207	1191273260
708	7-Ketocholesterol	44026840	6485712654	37550865	41232488	6426105498	9573949675
709	Lauric acid	66233007	94681151	13143552	12260728	19298818	12712662
710	Natamycin	2355224	39906812	10602338	3957566	9694093	1997091
711	p-Mentha-1,3,8-triene	1240650	14782253	1486984	1316509	52068718	25089795

712	Vitexin	648183	4067539	501768	875504	28637223	48705214
713	LPE 22:2	460473	46614265	479687	500936	42422099	18250772
714	Ornithine	59627513	8344654	63221052	59810554	1915723	1479060
715	LPE 24:2	90350	5970164	84242	82513	4007860	949951
716	1-(2-furyl)pentane-1,4-dione	9706237	7145466	21028895	65275699	600383929	1092925902
717	GlcADG (13:0-17:0)	156938	1108091	152334	152096	6459834	1841216
718	20-Dihydro 6α-methylprednisone	693801	5051932	542395	405186	13046196	52327550
719	4-(3,4-dimethoxyphenyl)-3-methyl-1H-pyrazol-5-amine	2699040	47524286	3751931	3029538	10790421	33730773
720	PE (4:0/18:0)	97048	6208371	92440	90294	3251152	364323
721	8(S)-Hydroxy-(5Z,9E,11Z,14Z)-eicosatetraenoic acid	429341	218538643	817903	689617	20635848	30551572
722	INK	2335297	45818208	2421022	3068310	11311855	11179421
723	Dihomo-γ-linolenic acid ethyl ester	73202206	114187299	77271283	59699643	596179565	669019131
724	LPC 24:1	101821	5584809	102770	100092	3773251	337918
725	2,3-Dinor-TXB2	113267	544163	470250	125120	6402834	475343
726	Diosgenin	239021	13346299	285867	281441	42663485	10617973
727	Quercetin	508568	391632	223430	343143	2191277	5961888
728	Pyridostigmine	190850675	77614085	199175031	280547587	224146540	341598510
729	Retinoic acid	113221	701121	120046	100780	6444438	2397763
730	N-Oleoyl Dopamine	175122	683080	177288	1638801	1701561	784776
731	PC (18:2e/2:0)	1510543	944259760	7752997	2569552	296348230	7791988
732	all-cis-4,7,10,13,16-Docosapentaenoic acid	3353782	218419142	2595111	1392303	72633216	109949851
733	Cycloheximide	711965	873284	553155	380852	1180160	6508750
734	6α-Naloxol	1626354	645915	1206647	1225264	313791	707153

735	tert-Butyl N-[1-(aminocarbonyl)-3-methylbutyl]carbamate	218819793	12914075	116834917	46396272	259945705	216536637
736	2,6-Di-tert-butyl-1,4-benzoquinone	5175834	34357420	10491636	11143451	34269474	1152018529
737	TNH	10535619	2630649	7988270	12297219	3971836	9100019
738	Indole-3-acetic acid	4633737	11664418	7368726	14200049	16833269	48293888
739	11beta-Prostaglandin F2alpha	820531	1148703	126373	47954	1305900	1913113
740	LPA 24:1	75035	2751864	64598	111385	6123059	2776360
741	PE (6:0/18:2)	231248	213297524	192838	193662	23480262	10823193
742	3-(1-cyano-1,2-dihydroisoquinolin-2-yl)-3-oxopropyl propionate	886951	4218599	1346923	793887	1261536	1102066
743	PC (16:0/22:6)	232020	205428983	463434	251884	68527338	1244342
744	Homoarginine	413187	650705	866558	1684597	531484	694444
745	ethyl 2-[(4-ethoxyanilino)carbothioyl]-3-(propylamino)-2-butenoate	454433	43047430	444601	527431	8834869	18279407
746	Isophorone	11474981	383744676	13786450	29738020	441689680	1103817436
747	Gibberellin A7	669709	411872	1588173	1429652	496850	5127466
748	DNK	4632777	11229602	2798756	13863307	4078889	2727031
749	Genistein	607886539	11042653	581042043	684251177	89924323	43944530
750	(S)-beta-Aminoisobutyric Acid	9365820	2464578	5458459	5187410	2152296	5495144
751	N1-(3-amino-4-chlorophenyl)-2-[2,4-di(tert-pentyl)phenoxy]acetamide	34304721	212899350	15224533	20235499	185756172	7867093
752	LPG 18:2	263938	2312589747	1570491	258761	24124057	25335965
753	Despropionyl p-fluoro fentanyl	8927526	1837530	11542875	10265411	5051699	5051849
754	Dl-Threitol	965997	1586523	1031666	1561054	2159298	2070946
755	Pyridoxine O-Glucoside	3590852	3555883	2508136	2133898	2400981	54244901
756	Phenylacetaldehyde	5051476343	114638369	475361071	2450163640	7781610594	9383931649

757	2-Oxo-2H-chromen-7-yl 6-O-β-D-xylopyranosyl-β-D-glucopyranoside	10401281	7954706	6610818	13249330	6818691	7923075
758	MLK	3537460	40746189	4477625	3871024	5739262	3270215
759	18-β-Glycyrrhetinic acid	1653675	176858832	1545708	1417623	7158156	2764529
760	Isoquinoline	203843321	79579096	19392113	151263607	14281292	18611781
761	3-Hydroxy-4-Methoxycinnamic Acid	1152016	1260129	1387961	1022974	533095	584577
762	1-Methylhistidine	6587229	5081496	10410326	2587098	10464705	20459047
763	Senecionine	2726757	18402385	3230298	2176916	17535026	46790116
764	Glycohyocholic acid Sodium salt	131184	658322	123267	149644	5217377	1998893
765	PC (17:1/17:1)	631855	34434048	3412891	4101465	25107480	3487114
766	LPI 16:1	313526	217364793	236525	257273	1863363	1380121
767	L-Cysteinesulfinic acid	781189	1922880	1281324	1246348	1543393	3290838
768	N-Desmethyltramadol	1440442	10928902	1693775	1570972	9457484	53090220
769	Raubasine	2351949	42190181	2795627	1916189	11186234	12074002
770	1-Palmitoylglycerol	665724163	1423594056	1196525768	918184650	9263767973	6647821670
771	5-Methyluridine	48518847	875258940	104845910	42747143	208057278	140891918
772	15-Keto prostaglandin A1	7587743	77594115	4359153	4557715	78896544	203083171
773	Pantothenic acid	24475691	44104059	16637407	15054049	977488284	765883295
774	FAHFA (19:1/20:2)	1097619	820014	116743	114716	950433	1285100
775	D-Glucoronic Acid	26349076	6711852	26779584	60330802	21089905	27623551
776	2,3-dinor Prostaglandin E1	10812675	168575538	14048975	18004666	155344125	196590560
777	HPK	8089553	28546409	9612245	10237794	8339363	22166911
778	PE (18:1/18:1)	480015	6205824	1187581	11225841	5357038	5559321
779	Biotin	36902826	38279971	39978404	64394636	33850713	44258853
780	Prostaglandin B2	240937	2791084	125805	204657	2221304	4255529
781	Tetranor-12R-HETE	2960557	7832953	3119977	2877800	45914352	34909035

782	23-Norcholic acid	1240914	1289383	143112	116542	867716	1294932
783	Dl-2-Aminooctanoic Acid	200743693	21526991	29466088	14075206	24743435	31984559
784	VMK	244203	35889188	292485	291122	4497186	1174979
785	D-Sphingosine	533190	1194334	16256436	62940326	898469	866345
786	Prostaglandin E2	355947	513949	510640	260628	3671295	386298
787	α-Eleostearic acid	130546228	8232565109	129695366	61018007	4752533943	6200747861
788	QMK	3506827	4625756	11109593	2621081	5319313	2781943
789	2-(14,15-Epoxyeicosatrienoyl) glycerol	2154292	536078590	2409611	2679063	212806229	1026977135
790	FAHFA (14:0/16:2)	1090898	560460	159988	93269	699784	521016
791	Gamma-Glu-Leu	8925651	13645534	8189555	7983882	9244945	8901042
792	LPS 8:0	495639	3689115	422233	472368	2308733	4323537
793	11,12-Epoxy-(5Z,8Z,11Z)-icosatrienoic acid	1984335	203256981	1226154	705029	75608541	38524215
794	PC (22:6e/9:0)	1162801	14489747	1460470	1242797	42986084	10274865
795	LPC 5:0	2471468	11263425	3267277	3402899	17446932	49546017
796	ACar 22:1	371707	36353316	364265	412685	44221506	28306219
797	Prostaglandin F2α-1-glyceryl ester	2542982	38564390	1713099	1508444	1669648	1301553
798	Riboflavin	27209147	36275873	5524747	3933558	109560654	932842753
799	glutathione	823893	537501	797940	848854	1397923	2253786
800	PC (22:6e/17:2)	270497	39281124	287140	372745	16628905	1142460
801	FQH	3617821	2069768	10015971	4984346	1489767	1370787
802	2-(3,4-dihydroxyphenyl)-3,5,7-trihydroxy-6-methyl-4H-chromen-4-one	1623838	3028423	1653888	1122810	3445287	48086521
803	Eicosapentaenoic acid ethyl ester	1303299	35816639	2156991	4387407	12570071	35390774
804	OxPC (18:1-18:1+3O)	134717	192766164	278691	165334	60164329	530913
805	Isolithocholic acid	1322261	9504369	1044433	1112728	4740244	44579921
806	13,14-dihydro-15-keto Prostaglandin E1	1854750	28200588	1199822	1790992	26491444	195695057

807	FAHFA (16:0/18:2)	20121024	13896008	8524256	5206155	101877675	197786286
808	beta-Cubebene	3548348	10517742	1914932	2021802	25516992	35847980
809	N1-(4-chlorophenyl)-3-(1H-pyrrol-1-ylmethyl)piperidine-1-carboxamide	641363	974942	660081	480665	3291408	3652049
810	PC (12:0/13:1)	1415868	36812311	2039116	1557357	19873999	4741087
811	Sodium [dodecanoyl(methyl)amino]acetate	2128454	15125263	4865854	4261429	38595073	15845277
812	Kynurenic acid	9756121	4474769	13868776	59565182	44301344	110007134
813	4-[4-(methoxymethyl)-6-piperidinopyrimidin-2-yl]-2-methyl-1,3-thiazole	8713502	7869665	6654269	10612646	27055815	10731365
814	KNK	1778895	35281878	1328301	697210	2894762	2541645
815	19(R)-Hydroxy prostaglandin F2α	7977200	132020656	6380917	5829634	72601171	205693967
816	Phenylacetylglycine	625251164	113564815	502057685	404769784	234010781	228261100
817	Glycyrrhizic acid	17596711	489109	45847536	894561	13816796	346334
818	N4-Acetylcytidine	2051400	27509098	2763965	2611629	23733052	44318028
819	DRH	3102060	4229035	8452784	2769948	8072803	1967391
820	PC (14:1/16:4)	379705	34786198	369094	380262	2640351	1994339
821	6-Keto-prostaglandin f1alpha	6019255	145312523	6186282	6237571	67364135	212165192
822	Punicic Acid	43178229	184351969	29791460	55207932	356832353	962586241
823	LPC 20:2	309327	190050429	764484	331699	44092020	2097760
824	Prostaglandin J2	5659204	97146785	50474293	54606293	133892986	935910119
825	4,6-dimethyl-2-(methylamino)-5-nitronicotinonitrile	1740493	738426716	2082956	2143746	59472377	277512491
826	Nalpha-Acetyl-L-Arginine	790595	934886	649272	645218	1458031	1078636
827	Butylmalonic acid	301373	1762129	301876	487654	2397657	2000737
828	N-(4-butyl-2-methylphenyl)-N'-[4-(4-methylpiperazino)phenyl]urea	13283777	192999717	14916241	11465642	551560604	966467856

829	LPG 18:1	177960	1875584504	10715996	1885503	138611538	84107276
830	LPE 22:0	416824	14456409	544485	528247	36079154	2739017
831	DL-Arginine	44731912	739567815	37659901	4608451	11784957	9924071
832	PQH	6484835	2461289	8271351	1782917	3558671	3778465
833	19-Norethisterone	1693206	32639018	2008224	2640657	34796732	34937687
834	Porphobilinogen	242131472	24183017	141737767	611742334	19508139	37775244
835	1-benzyl-4-{2-[(4-fluorophenyl)sulfonyl]ethyl}piperazine	2866101	20435689	3459826	1234296	3600296	2192249
836	ARH	4435513	6739571	9362197	9831748	2188560	4588558
837	Methionine	11955065194	530247279	214370621	505145426	91357564	69042626
838	ELK	24311040	764076046	21405720	22920676	443858779	498352183
839	Limonin	13590234	65758843	20054164	13031437	409310590	961331233
840	7-methyl-3-nitroimidazo[1,2-a]pyridine	163403958	21560846	204282134	2875438	115549297	3555052
841	Sedanolide	31336933	112573688	68359076	30908674	179931719	959157547
842	1-(3,4-dimethoxyphenyl)ethan-1-one oxime	5114746	30812869	3276932	3852289	24291886	26624221
843	PC (17:0/17:0)	1310922	28703807	604750	460835	8729553	1486221
844	Adrenosterone	551576	441737	231873	267597	1476424	1467055
845	N-cyclooctylurea	5194744	44812619	6765503	11902623	273965904	945404417
846	LMH	4139417	29309763	2056283	2140595	5040125	2207030
847	Cotinine	129259850	14348605	183818389	167459903	83162218	15837703
848	LPS 12:0	115640	622275	104665	66048	1110848	1101846
849	11,14,17-Eicosatrienoic acid	1391700	32197689	958850	899851	5304272	9803682
850	2-Isopropylmalate	424277	552906	240460	204756	405219	651422
851	Obscurolide A1	169881702	17402310	59105128	107267709	20881138	17963070
852	Adenine	1191371181	14189599289	688355973	188420104	874248678	706451395
853	Hydrocortisone	78692	1089074	74283	77149	744636	343433

854	Glycitein	98179988	7080673	129517649	212245368	56723247	11822811
855	N-Acetyl-L-phenylalanine	247392438	79718148	342742477	631465559	214064284	283271245
856	2-Hydroxycinnamic acid	44095294	6878472318	96569503	138124646	239840319	78416607
857	8-Hydroxyguanosine	90892	92707	65875	59076	50256	900646
858	Emetine	4379112	742160732	7418512	4128413	274107750	24081062
859	N'-[4-(tert-butyl)phenyl]-N-cyclohexyl-N-methylurea	1539924	2849326	2113386	964542	6516675	35308011
860	Metanephrine	3313224	28293093	3999572	2472215	14841300	38873826
861	N,N-Diethylethanolamine	3317671	5364378	3289445	8339307	24130161	38368513
862	4-(4-chlorophenoxy)-3,5-dimethyl-1H-pyrazole	75386379	365261237	257738387	1695292473	5384742000	7408292450
863	Pyrophosphate	70492243	1956019789	7257999	45117576	695876281	515801543
864	PC (16:1e/22:6)	2358617	701551375	2575215	2110917	478024318	7182784
865	LPG 16:0	20423650	1919989626	4882688	1656385	145183520	37498797
866	Estradiol benzoate	4376550	2214012	3591213	8035701	2344322	1442288
867	SM (d14:3/12:1)	1814327	27415968	2375643	1592340	8858735	3268824
868	Tretinoin	11071827	42740177	21910150	25152887	148701902	173892077
869	PC (9:0/13:1)	345384	28950120	369981	384173	27543171	13035787
870	Salicylic acid	5403118	159987327	2632153	2427061	84160449	66897991
871	SLK	2008876	25612159	2374596	2599308	13813032	2093055
872	LPE 19:1	603352	168810698	15912735	969168	36811323	8463994
873	SNK	2270720	24522028	2427686	5154863	16686704	2620295
874	Solanidine	525833	22959590	735464	528036	30523108	28840513
875	N-Isovalerylglycine	563652872	51131934	466608767	376654542	121555727	91577139
876	1,2-dihydroxyheptadec-16-yn-4-yl acetate	14017061	62948965	19229671	11490220	85795379	836174142
877	PC (18:4e/16:2)	1302074	11230411	1899649	1789262	31925858	24906301
878	4-(2,3-dihydro-1,4-benzodioxin-6-yl)butanoic acid	61160615	38147458	104538392	207753769	104150266	98310441

879	gamma-Glutamylleucine	420918426	52125952	1318171483	17788383	427385932	261134240
880	LPC 13:1	6126122	3201192	2279128	823737	2814295	2474735
881	6-(3-hydroxybutan-2-yl)-5-(hydroxymethyl)-4-methoxy-2H-pyran-2-one	3767521	5779404	3837241	2605070	5873138	30836107
882	Citrulline	30154655	4222185	40751906	46151824	5000489	3814325
883	Monocrotaline	1292279	2988694	1654186	1535144	28079925	11685700
884	(±)8-HEPE	527507	26807340	401341	359882	22643659	29267959
885	AKB48 N-(4-hydroxypentyl) metabolite	1880935	24431057	2008899	2082416	2759365	1932355
886	3-Indolepropionic acid	5964753	7318048	7447312	5908960	7747920	11073880
887	α-Estradiol	47488022	15302209	26330191	25306568	32916933	22869199
888	Chenodeoxycholic acid-3-beta-D-glucuronide	2083630	2057082	7109185	1438336	3430297	2202897
889	Mestranol	160410081	319573181	334571986	519798124	1972694735	1820338660
890	N-Acetyl-1-aspartylglutamic acid	44088285	2273270	36066942	44491609	4913192	11491324
891	PC (19:0/20:5)	1211945	646075039	3117812	2008455	406943732	5394554
892	VNK	4537394	1905040	7054833	1710584	1808834	1351845
893	Linolelaidic Acid (C18:2N6T)	1418528	23301784	3362477	4258762	18778858	28635067
894	Ofloxacin impurity E	46154737	2621264	18015648	24151456	4295233	3798426
895	LPE 17:2	1235189	25820317	781411	960171	6056105	5916654
896	4-acetyl-4-(ethoxycarbonyl)heptanedioic acid	156763092	11545086	108286007	199993355	40897389	30342057
897	1-[(1-benzylpiperidin-4-yl)amino]-3-[(4-fluorophenyl)thio]propan-2-ol	155858920	7849823	63811812	113261720	50495615	58589395
898	Vitamin B2	3309528	11632426	4522685	7128961	10655646	29619529
899	LPI 17:0	110026	153309119	101624	103008	16140684	969037
900	LPE 16:2	878375	23033093	819647	787828	20725236	12265223
901	LPS 14:0	397117	156187025	420230	415454	78719646	6193178
902	Glycerol 1-hexadecanoate	551732	15600053	490774	771030	10236541	26031436

903	2-(acetylamino)-3-(1H-indol-3-yl)propanoic acid	158363733	8596801	156789433	189293605	40026779	68514749
904	PE (6:0/18:0)	180413	158098141	109115	121201	83165027	3108181
905	LPC 20:1	384633	134401221	758787	417394	70169633	1935204
906	Reserpine	12996032	901819	4998917	48458133	1326506	4702082
907	OxPE (16:0-18:1+3O)	738683	1877010731	1841501	1228188	837655285	5773326
908	3-[(methoxycarbonyl)amino]-2,2,3-trimethylbutanoic acid	44837222	3154130	37485318	42021804	3701285	21473362
909	Anthranilic acid	8022257	64892822	138622724	2032653740	39905470	1642608984
910	PC (14:0e/7:0)	753716	24486123	1190507	901997	26723060	3182431
911	RPH	27586341	3779248	182735159	10229591	20324024	4144267
912	PC (16:3/16:4)	886393	19662164	960467	839860	5765737	5039997
913	(2R)-2-[(2R,5S)-5-[(2S)-2-hydroxybutyl]oxolan-2-yl]propanoic acid	235179	2244427	249601	359301	24181826	6294690
914	PEtOH (18:1-18:1)	152862	15654757	137148	133133	149539849	133345481
915	PE (18:1/18:2)	524371	21875095	435794	4036032	17908083	1424453
916	HNK	2736038	12275052	5916921	1541532	5432681	1191982
917	TPH	1909908	23652322	2210052	2072047	25152988	11153442
918	PC (18:3e/2:0)	2338468	630856776	12245407	2964728	232819375	7308982
919	Glycyl-L-Proline	42092468	34549933	25349156	16255497	16182897	17032460
920	4-[2-(trifluoromethyl)phenyl]-5-undecyl-4H-1,2,4-triazole-3-thiol	8424480	122847527	14097836	9242517	3855982	3092135
921	EMH	3300172	5431109	4299690	3371513	17169645	27025130
922	Daidzein	396648000	4014534	442484586	520407015	78727844	64824451
923	3-amino-2-phenyl-2H-pyrazolo[4,3-c]pyridine-4,6-diol	8923223	75143514	27896758	35401846	291830729	781333983
924	Thymidine 5'-monophosphate	1611085	134802909	19526393	534537	3623608	11200560

925	3-hydroxy-3-(2-pyridylmethyl)indolin-2-one	5280189	8299131	3001211	6287773	12106036	22715620
926	JWH 018 N-(5-hydroxypentyl) β-D-Glucuronide	38236392	1409110	42652431	41831928	5519066	2961904
927	Bicyclo prostaglandin E2	80713182	669565905	112614169	202433288	2051085650	7399401220
928	3-Methylhistidine	5085618	1942652	1911813	4052265	2433931	2643961
929	Homovanillic acid	1512449	4231374	494109298	316324013	6564412	223740998
930	2-Isopropylmalic acid	5239616	1185374	35993740	762501	743632	5509697
931	L-Lysine	2012210630	85879558	3431641272	41574824	397106956	227224324
932	D-Threose	42584205	11634444	31712389	31442630	31948606	35001752
933	PC (13:1/18:5)	201187	21078657	489715	228967	12196730	1573814
934	2'-O-Methyluridine	36866529	1982938	13249810	2132306	2555808	21506470
935	Dl-P-Hydroxyphenyl lactic acid	17230533	28452844	20497142	19838211	103773347	724177430
936	Prostaglandin K2	49529494	193597898	53752081	38127454	368347109	761985435
937	10-Hydroxydecanoic acid	11136489	87874391	14178075	25208121	121489282	162256739
938	cis-gondoic acid	3475970	20728817	1315011	1087705	9526567	11037733
939	PC (8:0/16:0)	138590	147477167	285030	229567	76601625	366385
940	PC (18:5e/18:2)	447698	17050575	644769	483011	7489090	1788822
941	OxPE (18:0-18:2+2O)	345825	129746753	336860	424039	80348648	1152316
942	3-[4-methyl-1-(2-methylpropanoyl)-3-oxocyclohexyl]butanoic acid	1316730	61359024	1006326	1115396	102852760	609533765
943	OxPG (18:1-18:1+3O)	882651	145545390	591330	476933	51681758	20549699
944	KQH	2567312	18323790	907503	1202109	746001	894235
945	Uric acid	20105186	1980380	2099743	931021	7416536	154028618
946	12-oxo Phytodienoic Acid	14295610	77441285	16773547	9808142	678686892	170806074
947	4-(3,4-dimethylphenyl)-1-thia-4-azaspiro[4.5]decan-3-one	1626146	5347770	1752742	1073177	13355433	22576232
948	10-Nitrolinoleate	23812352	122162015	34737779	81534476	268738111	640686381

949	PC (16:0/16:1)	644229	537144265	889549	1055582	370405169	4857237
950	N-(2-morpholinophenyl)-2-furamide	4165693	3687002	4415243	5403346	1803935	10974782
951	(2R,3S,4S,5R,6R)-2-(hydroxymethyl)-6-(propan-2-yloxy)oxane-3,4,5-triol	2038094	12581751	2134366	5120386	1983448	2099265
952	3-Acetyl-2,5-dimethylfuran	1034319	7457776	943758	899662	21641300	8271794
953	Pentadecanoic Acid	643672	101496765	1389217	1922547	36033304	25575296
954	Amphetamine	4031429	12840143	4956047	4071373	6610070	6459103
955	4,5-Dicaffeoylquinic acid	24708692	41256379	43687327	49438452	2349676764	5907032489
956	ACar 17:0	708065	19207492	529933	500178	8149774	5201873
957	GNK	956989	17812688	1613317	2716804	1283760	1035643
958	Vitamin A	399365	9134872	575069	483598	18491856	18201395
959	MGDG (2:0/8:0)	8146414	38191151	9395461	12106906	7599991	152584747
960	Tomatidine	546051	572003184	1241596	1462443	401315943	427246686
961	1-Palmitoyl-Sn-Glycero-3-Phosphocholine	5692467	1680817925	21312988	2329525	210758155	11862433
962	Lanthionine	1188996	5816431	796566	5238070	142285468	41562078
963	4-Nitrophenol	2284056	135120428	4218397	3611147	63888173	71532730
964	Dl-Glutamic acid	42619420	11611072	148008295	2939482	11128214	13099686
965	Oxolamine	4277861	5530044	2572068	2397021	2838659	2201103
966	TKK	1568384	17443666	2252941	1843928	5609326	1781093
967	Paracetamol	38223819	16860529	38206188	40046141	18965638	20229649
968	PC (20:4e/3:0)	469704	17633063	549264	599703	17069612	12295306
969	Nervonic acid	1770189	4214620	1514765	1715111	49098516	105611460
970	8-Hydroxy-2-Deoxyguanosine	3975885	4998561	2731295	1579932	11277199	5617025
971	YPH	3041717	1515515	3615817	4191707	1628817	1022047
972	Tetramethylpyrazine	134232090	34498724	128876173	145861960	42626552	47616970
973	PC (9:0/9:0)	178516	16165776	201099	208612	3953923	1431850

974	Prolylleucine	1296356532	115454940	15359770	22872108	21609833	19553830
975	(11E,15Z)-9,10,13-trihydroxyoctadeca-11,15-dienoic acid	9004364	209489297	12917864	18163445	159488911	703169225
976	(R)-3-Hydroxy myristic acid	48381154	49599301	147722465	65283632	1769104197	92688555
977	tetranor-12(R)-HETE	683935	898139	466791	210563	145729398	30914369
978	5beta-Androstane-3,17-dione	726974	9117415	685319	911167	17043342	7525011
979	2-Hydroxycaproic acid	40408399	154539732	2536625052	2658874530	116754853	198013523
980	2-Hydroxymyristic acid	47858741	47897246	596292	1380544	1761826022	87276662
981	TMK	30888424	540257705	46108855	22463036	11781339	3939818
982	17α-Methyl-androstan-3-hydroxyimine-17β-ol	528174	10110577	607624	596250	16322978	17772269
983	N1-[1-(2,4-dimethylphenyl)ethyl]-2,2-dimethylpropanamide	3539120	1840644	4165401	4211822	2448773	9412511
984	KPH	2043646	10793922	1947800	4623846	6953459	2109686
985	4-Methylphenol	2813025	4179127	3123669	37277063	10098583	76086014
986	Pro-Leu	22726193	10901434	13469436	9992122	5140358	5249214
987	1-(3-methoxy-2-nitrostyryl)pyrrolidine	2065485	14808335	3476390	3763450	5878900	5733317
988	Azelaic acid	949357	34956723	2072556	1792425	16961110	143126770
989	PB-22 N-(4-Hydroxypentyl)-3-carboxyindole metabolite	518353	1482424	787063	4564782	1850379	1345656
990	15-Deoxy-Δ12,14-prostaglandin A1	51047988	126870908	29474103	38139991	154396035	640949733
991	N'2-(2-hydroxybenzylidene)-5-nitrofuran-2-carbohydrazide	282311	4074467	330940	491606	12119912	16941695
992	PC (20:0/22:6)	180760	12088346	209997	231081	15812731	1374467
993	7-Methylguanine	130307604	90260048	131846090	169609323	49330080	227329377
994	OxPC (16:0-18:2+1O)	203485	118332120	297320	196545	37498109	608504
995	Indoleacetic acid	9945836	3762455	20824760	37357452	11703313	25739889

996	LPE 17:0	1602725	556373598	4101630	2158424	428467616	23005764
997	N-acetyl-L-ornithine	461299897	8753500	179979180	257348731	24334528	24568636
998	GLK	975923	13139840	1240900	2283077	992312	1110074
999	1-Stearoylglycerol	1050611382	788501459	1035399993	824108965	6190216796	6618367979
1000	ANK	1718442	13020289	2557756	1816790	6526140	1559456
1001	PC (18:3e/22:0)	134006	10998881	148534	158546	9974035	15842693
1002	Irganox 259	1445955	9108011	296998	293985	3060719	2305271
1003	Rifampicin	30241290	1513333	6253364	35125149	3049904	907580
1004	PC (22:5e/18:3)	929659	552258176	1702017	528541	318142694	3709318
1005	Gly-Tyr	2306122	1542545	1036687	1261296	1980825	3132951
1006	9-Hpode	462987	124943750	425716	400411	58288351	52819174
1007	Cinchophen	646476	5916023	643400	873453	9927437	15778989
1008	Glutaconic acid	6636282	16497182	15241519	12911137	118597545	100435996
1009	Docosanoic Acid	12447915	23207196	11119349	11013197	63020726	107505050
1010	N-Acetylvaline	450887937	79164451	260389989	217691856	93261118	92907599
1011	Nepsilon,Nepsilon,Nepsilon-trimethyllysine	1560637	13735141	1905033	1736484	6647135	6262808
1012	FAHFA (18:0/20:2)	3825489	956296	1879937	1867315	22372065	110181632
1013	Guanidineacetic Acid	3220667	3834302	3383567	2795185	7997304	6196219
1014	RMK	1160382	10715991	1442669	1739726	13356085	2558279
1015	LNK	1333586	13399222	1627715	1932051	2222542	1291154
1016	FRH	1783214	471343757	2949238	1704682	1848538	2330210
1017	2'-O-Methylcytidine	7227826	788254	35192739	5154727	762704	676644
1018	IMK	3082320	12355527	3726475	2146414	5348686	2807472
1019	D-Ala-D-Ala	34409577	5106038	19459812	12522455	6312457	10119382
1020	Ouabain	347748959	3190891	431695742	433009200	55389993	4359835

1021	5-(hydroxymethyl)-4-methoxy-2,5-dihydrofuran-2-one	1186592	8573983	1257026	1349943	8152409	13236751
1022	Uridine	35166557	28385315	19212285	10677990	24502766	61357829
1023	geranyl pp	2535370	3623932	2723910	2538853	3234854	16236157
1024	OxPC (18:1-18:1+1O(1Cyc))	200836	120209398	270308	186454	39411496	571741
1025	2,4-dihydroxyheptadec-16-en-1-yl acetate	4936126	179018005	15556210	13566176	111916441	634598216
1026	Tetrahydroaldosterone	2525444	4161489	3441106	2883842	8480717	2808258
1027	Theophylline	1545533	4546559	1849644	2660674	66424317	97585786
1028	α-Phenylacetoacetonitrile	33753409	15205490	26559072	26105659	13206378	14181655
1029	L-(+)-Tartaric acid	1716676	10467967	3387528	19945096	791297968	1198616317
1030	Cyclo(glycyltryptophylprolylglycylvalylglycyl-β-hydroxytyrosyl)	1352863	12669810	1569818	1550818	6170457	1382908
1031	Undecanoic acid	18084875	28517936	33136295	21412720	131231003	18960508
1032	C-12 NBD-dihydro-ceramide	464815	2420945	430674	470410	12107307	8911793
1033	L-Methionine	3112711926	320181743	87733309	162685601	36236791	22771232
1034	PE (2:0/20:4)	345402	116392037	227918	230322	2471604	1049532
1035	PC (5:0/13:1)	5240529	5198284315	11834208	2582856	272873106	32084229
1036	alpha-ketoisovaleric acid	412918231	109527003	318042382	259594928	87404029	134736150
1037	Asp-Phe	30364741	6673641	10507290	7595980	2970207	4863871
1038	MKK	1423164	11450237	1682254	1760239	1881089	1475507
1039	4-(4-cyclohexylphenyl)-4-oxobut-2-enoic acid	33426434	20544005	3277502	14061995	23927225	28235095
1040	PC (13:0/14:0)	426648	9907825	528027	485331	13014356	4048272
1041	RLH	182280	7125952	242048	251146	1746323	200567
1042	Di(2-ethylhexyl) phthalate	1731357	2345993	2076735	1802523	11606576	11186051
1043	LPC 16:0	4147381	1442063884	15497466	1367701	126543129	4175449
1044	L-Tyrosine	60887977	5336122483	113677877	144582668	220209638	112827901

1045	5-Hydroxylysine	63714670	19999910	107870468	143494113	24846148	85976569
1046	VLK	3900921	476849572	2447759	2308996	1546057	1183667
1047	L-Proline	1131485356	592992566	234375540	903878077	403754460	474393109
1048	Valylproline	104718060	17443654	11686755	13002664	17489211	25659673
1049	Dihydrouracil	82039692	103924242	86508744	73078832	256770451	588080014
1050	12-Hydroxydodecanoic acid	30029100	5309145	9670875	4374116	16610802	74452622
1051	11-Deoxy prostaglandin F2β	1401893	17569217	1522207	2339546	32718825	118576752
1052	FAHFA (18:1/20:3)	201356	41709431	138193	82591	39269635	107756178
1053	N,N'-di[4-(2,6-dimethylmorpholino)phenyl]thiourea	87400895	6788515	102415226	142625434	38647050	39110682
1054	cis-7-Hexadecenoic Acid	2792153	67868412	3203352	3842627	106674569	555711114
1055	WPH	1086253	9898490	1546638	1182063	4557525	1126550
1056	Homosildenafil	82364174	2313340	116883155	73189213	29667459	139781057
1057	5-fluoro AKB48 N-(4-hydroxypentyl) metabolite	116793015	4915959	12754891	1780395	20783299	4319174
1058	3-hydroxy-2-(3-nitro-4-piperidinobenzyl)propanenitrile	735163	9048145	886169	365644	2426083	2873141
1059	3-[(4-hydroxyphenyl)methyl]-octahydropyrrolo[1,2-a]pyrazine-1,4-dione	1251758609	332497951	1327035849	1667926527	1883000717	3051452148
1060	RQH	762121499	52446281	745888839	1412419112	155736325	76202530
1061	3-hydroxy-2-octylpentanedioic acid	3266641	14878457	2839774	3823180	15314731	600576840
1062	OxPE (18:1-18:1+1O(1Cyc))	172073	100115623	173410	197956	39077169	527506
1063	L-Phenylalanine	35722471244	9298681755	3800431625	17792198159	2761501935	1788607417
1064	L-Histidine	2210019228	86245457	1910248667	7099789	221123186	14734585
1065	PC (9:0/10:0)	332784	8803889	377149	472992	7078990	2532390
1066	GKK	611483	8436342	780421	811064	2571322	674985
1067	D-(-)-Fructose	404947466	243574022	340377418	315080156	234671440	294753851

1068	OxPE (16:0-18:2+2O)	271282	108420630	302579	375302	101759764	1756958
1069	Deoxyinosine	1669982	7925777	2216939	1977136	5118158	9078390
1070	N1-(1-benzyl-4-piperidyl)-4-chlorobenzene-1-sulfonamide	4050301	36787041	5910362	27877850	427730967	545303834
1071	Eicosapentaenoic acid	5436703	486608984	4088859	2698031	140505887	140020134
1072	PNH	850242	1402232	1145544	2808049	1037570	3538066
1073	IMH	954344	4251417	920985	2709296	973250	1102456
1074	D-Xylitol	10851193	11062316	25167727	17455326	62924962	94274040
1075	DPK	12770055	5031096	53447907	135566639	10725377	3954623
1076	YMK	710081	7553194	922846	693391	4734941	9691983
1077	Jasmonic acid	22098032	56348857	17201682	30907125	68862739	57502238
1078	Palmitic Acid	1225056471	4406089995	734425421	678636041	5636409739	5783633719
1079	N3,N4-Dimethyl-L-arginine	83047894	101739738	105456431	132636818	96823242	207962226
1080	3'-Hydroxystanozolol	2788939	111312674	1594226	1792926	1126082	1230802
1081	Mirtazapine-d3	43331561	3018184	73474688	115027446	21054040	31618728
1082	SM (d14:3/18:2)	298465	2531502	290293	307858	8956447	2030963
1083	9,10-Dihome	2145465	8070064	3901556	6792288	8955345	580657757
1084	LPE 22:5	641853	387945178	1528421	683093	68379572	5463598
1085	PC (18:4/20:5)	545433	7074747	804646	625509	4326408	1711424
1086	PKK	1475659	4164994	1933409	2249201	860841	1152982
1087	N1-(3,5-dichlorophenyl)-3-amino-3-hydroxyiminopropanamide	392379	3039520	367753	349530	5368681	9004968
1088	4-methoxy-6-[2-(4-methoxyphenyl)ethyl]-2H-pyran-2-one	7906448	38813470	12017861	33489360	356681313	483714247
1089	MNK	1252611	6141944	1539901	1349747	1987594	3596727
1090	3-Methoxy prostaglandin F1α	2852567	71573384	3513345	7583697	112855658	551579695

1091	PC (16:1/16:1)	204836	6109488	217173	216673	2634986	1438990
1092	cAMP	15110093	106060740	6841266	2494540	40014931	7306551
1093	N,N-dimethyl-9H-purin-6-amine	58976291	14389145	9587526	1008209	128448559	4839395
1094	4-(4-methylphenyl)-2-phenyl-1,3-thiazole	890118	5189210	961607	753667	6904137	4619252
1095	L-(+)-Citrulline	66449038	7982409	115180404	80460320	2047143	1866127
1096	cis-4-Hydroxy-D-proline	33932149	18726292	12942884	114429760	73326918	427095238
1097	Pilocarpine	1107470929	78982003	700031412	1239141478	84563269	178664990
1098	(+/-)12(13)-DiHOME	190027699	491078747	157603257	95218148	1056806492	1316289634
1099	LPI 20:2	98632	106002557	92284	89390	2199772	905500
1100	cGMP	602226	3061337	683747	702231	6593555	2668369
1101	17alpha-Hydroxyprogesterone	1079935	1708223	1638235	934938	1483460	1154698
1102	Mesterolone	170267	5468499	201289	194701	2573760	1352336
1103	PC (14:0e/3:0)	9067088	5101295990	41240435	9553478	1251663196	28382013
1104	cis-2-Decenoic acid	30448081	14442158	41148419	25269149	1452304200	311781155
1105	1a,1b-Dihomo prostaglandin F2α	4331287	12896364	2962617	2065585	53659305	104377055
1106	DL-α-Aminocaprylic acid	4648672	422561297	3380458	4187666	240490064	362798109
1107	Sildenafil-d3	10334278	710635	11402190	24369448	1768096	657994
1108	D-Arabinose	24967548	6433830	26851754	2924181	22388490	15574253
1109	Bilirubin	16811546	563849	15139514	27352357	1367283	1211691
1110	methyl isoquinoline-3-carboxylate	513026	4478204	744589	508643	3947955	5383162
1111	4-[3,5-di(tert-butyl)-1H-pyrazol-1-yl]benzoic acid	28127284	1276461	1228740	1455629	2052166	1451919
1112	dCDP	583552	3982209	599455	551826	1610843	1991584
1113	5,8-dihydroxy-10-methyl-5,8,9,10-tetrahydro-2H-oxecin-2-one	102836408	8054880	1155780	1607646	3423313	4989925
1114	PC (17:0/18:1)	109700	3933266	119405	131932	3452008	1473155

1115	1-(2-naphthyl)-3-tetrahydro-1H-pyrrol-1-ylpropan-1-one hydrochloride	118753	3626931	141129	144813	1409237	574621
1116	O-Benzyl-L-tyrosine	483617	2632887	431823	892995	2087372	527249
1117	PC (16:2e/16:3)	130879	2192038	198521	173150	3395688	601508
1118	Succinic acid	2562373466	812807488	2013696128	2770597367	164991510	233729852
1119	2-hexyl-5-(4-nonylphenyl)pyrimidine	1450691	38781480	1779886	1246570	67050248	418127068
1120	α-Linolenic acid	36616315	198501528	21460545	21402282	436810365	5922140756
1121	LPA 18:2	1171377	1199818266	6200134	878404	24894318	22923086
1122	Thymine-d3	337165	1809991	355245	343371	1764648	1281094
1123	N-Acetylputrescine	349798278	75206395	578548708	1323531346	514228387	1343943587
1124	Octhilinone	126086	1976460	188120	235579	1727163	866125
1125	L-Threonic acid	2583464301	1407041886	2022465865	2761406114	152303558	224998958
1126	PC (17:2/22:6)	644750	423256364	1001494	719508	62672717	5673408
1127	JWH-018 N-(3-methylbutyl) isomer	99649604	4969981	9699023	20350460	23397581	24482512
1128	PD 0200347	25246471	2805799	50661053	104171504	4875537	14366595
1129	N-Methylthreonine	4522928	2158563	24982505	18084983	7341370	19870378
1130	4-(tert-butyl)phenyl 3,5-dimethylisoxazole-4-carboxylate	96284655	4749161	22412181	20745848	5716217	8695314
1131	17-AAG	26530692	963465	19665282	440038	1465569	1017835
1132	2-Phosphoglyceric acid	3543491	59168782	27652095	17358970	38923249	70809743
1133	DL-Malic acid	15139960	7280314	364165283	75930958	52482830	12932992
1134	LLK	1686843	371331879	2230005	1943019	3102963	1784001
1135	Heptanoic acid	1280162143	8434688	2526399633	2290964335	220510403	303040798
1136	2,3-Dinor-8-epi-prostaglandin F2α	25629531	793068	23469524	399923	3133727	856825
1137	LPE 18:0	14157606	4713203574	33647983	30110955	4690607132	783885817
1138	N-Acetyl-L-leucine	48075339	47321848	41639643	123093674	165797512	354750246

1139	N-Acetylsphingosine	148047	90498102	138601	110615	38296771	8184325
1140	FAHFA (18:0/2:0)	1753939	78259486	1913921	976388	24813968	58059557
1141	Citramalate	4393578	7757517	14855337	69282338	371388849	461748606
1142	RKK	371575	337910186	326120	243303	1819048	1772788
1143	LPS 19:1	141436	90912649	163977	130253	7735315	5186516
1144	OxPC (18:1-18:0+1O(1Cyc))	262906	83232790	326535	554985	18656078	975652
1145	11(Z),14(Z)-Eicosadienoic Acid	9362051	455691601	5626061	6236343	452150980	1096202908
1146	Spermine	11386378	3332056274	36113961	7892345	1496203413	3729654107
1147	Methionine sulfoxide	603002924	2303607924	320719614	390605869	175628001	129623691
1148	Pyridoxamine	65717763	104143854	100063337	118748888	155623684	137945981
1149	LPS 20:4	286961	87744393	356885	247737	24018593	3501996
1150	6β-Hydromorphol	22335556	1633458	17180841	14836416	2673383	8150187
1151	trans-Petroselinic Acid	3071468	22625998	2571391	3652992	53013163	81438456
1152	Docosatrienoic acid	1277137	28153298	634271	1374287	23792478	80896387
1153	PC (2:0/16:1)	356502	940225157	700618	269027	26297432	1260459
1154	Ala-trp	24151573	1915093	16253077	20367980	14202573	17337847
1155	OxPG (16:0-18:1+3O)	745200	87782355	478891	376094	38732175	3379284
1156	PA (18:0/22:5)	134342	88003927	163771	155825	69860104	462295
1157	(±)5(6)-EET	5300690	91428439	4141642	6203595	190477352	438121885
1158	PC (18:1/18:2)	6677284	27486284	72703587	96241292	26750879	23836335
1159	N-Glycolylneuraminic acid	670876	80420541	263827	404452	611335	912961
1160	LPA 20:3	283804	81939209	289746	167682	2351135	1661556
1161	2-{1-[2-(4-benzhydrylpiperazino)-2-oxoethyl]cyclopentyl}acetic acid	24330042	551674	10260902	9651429	2076485	1910116
1162	2-Hydroxyphenylacetic acid	1687817	4019179	2372547	25009286	7711714	53143776
1163	Caprylic acid	317805840	269451038	222799427	216475374	931817215	132101107

1164	Tylosin	20164974	733677	22288471	3342786	694355	720591
1165	Histamine	85282909	76906336	8920720	10908768	75327008	69392396
1166	13Z,16Z-Docosadienoic Acid	2584090	14594656	702564	951048	28638537	76497847
1167	PE (4:0/5:0)	7600314	348358382	8811961	8819032	186572357	353053264
1168	Isopentenyladenine	23884582	3999740	10464843	312886	9654941	37443227
1169	D-Glyceraldehyde 3-phosphate	7322586	10814855	11213122	11957637	28998831	91245281
1170	9-Oxo-10(E),12(E)-octadecadienoic acid	22494990	28805589648	111214162	58412258	4315988319	8304515819
1171	LPE 22:3	128048	78655725	207321	147892	26433912	3236564
1172	D-Fructose 1,6-bisphosphate	933035	9950872	8810293	686344	28361403	90475802
1173	Thr-Leu	82025313	10770772	81053561	107443508	40137811	46707594
1174	N-Acetylornithine	303428503	8146723	128462339	104775742	39956913	16886114
1175	ANH	1548796	6334979	1407537	1055710	14670242	448587201
1176	TLK	2886209	309469838	3619115	6407354	3646816	4156212
1177	FLK	3627745	349190260	4867951	3910896	7444183	3584384
1178	4-morpholinobenzoic acid	1524958	1905181	316952476	30741386	2011992	2615279
1179	Delta-Tridecalactone	3705968	8084459	6534346	3983845	87361753	39601029
1180	N8-Acetylspermidine	4512806	11771803	14764730	55824256	404404890	205905306
1181	4-Oxoproline	2430627462	746699088	2106583281	60729023	545525226	221756616
1182	Choline	4166823384	11947551368	5480968768	6766659428	17569046807	27623354811
1183	Mevalonic acid	57334421	6791015	69714733	48070238	466959739	1130031790
1184	Thromboxane B2	81326455	3649962	87191781	5934606	28048403	48217946
1185	HRH	85324634	86111209	78381973	89312950	192675792	238487921
1186	T-2 Triol	66813639	7194598	84069794	37939230	7833184	5494447
1187	Amikacin	43690838	5281522	98279573	58323119	17066227	2163210
1188	D-Galactonic acid	8687667	15339373	14542281	64854272	362625533	420038502
1189	2-Amino-1,3,4-octadecanetriol	6863210	4401452618	13101635	10166298	2565240626	3198522643

1190	Avocadyne 1-acetate	7117141	45804436	7055378	4994088	203230727	428856689
1191	13,14-dihydro Prostaglandin E1	8701432	7906264	1706079	1188558	71733862	4133639010
1192	3-Hydroxybutyric acid	5470144	13417806	4994613	12310598	11899191	85028969
1193	15-Deoxy-Δ12,14-prostaglandin J2-2-glycerol ester	83320403	4645413	87206141	16561390	21467416	13717066
1194	1,4-dihydroxyheptadec-16-en-2-yl acetate	5921908	40614883	17312033	18061785	125961337	397695881
1195	Prostaglandin F2α 1-11-lactone	1240054	80591965	979101	909410	35884495	55032115
1196	8Z,11Z,14Z-Eicosatrienoic acid	6075177	1053669248	5643919	7722756	605840113	882438544
1197	Estrone	80541748	155992591	50105591	64192330	64727751	69326869
1198	4-[2-(2-methylphenyl)-2-oxoethyl]benzamide	1246512	1675760	1280136	1079240	4169255	80406531
1199	gamma-Glutamyltyrosine	82514105	2614737	93142614	2229433	19248029	5182983
1200	Pipecolic acid	686492200	331490977	1137812535	293244919	497820150	690594635
1201	Decanoic acid	226846215	169715466	178122214	113099559	1135692576	163614714
1202	2-(3,4-dimethoxyphenyl)ethanamine	22254842	4743118	10863678	12846917	8010545	7574365
1203	(6E)-7-(2H-1,3-benzodioxol-5-yl)-1-(piperidin-1-yl)hept-6-en-1-one	4146616	334151630	2983725	3858891	5724002	14033358
1204	Levalbuterol	81698234	17746516	36743776	48339794	29128785	39508511
1205	N2-Methylguanosine	21935055	593525	1500729	9516646	1883744	2451135
1206	5(S)-HpEPE	279996	4430082	320843	305110	76544547	4864519
1207	Gly-Ile	30166307	13096527	68032837	56558884	20913006	23087907
1208	LPG 20:2	109234	75097837	123389	100598	14924294	3677290
1209	LPG 18:3	175475	39743155	198649	186360	6679221	1169293
1210	3-[2-(1,3,5-trimethyl-1H-pyrazol-4-yl)hydrazono]pentane-2,4-dione	15599645	49572206	13625472	22918400	15705104	25901007
1211	Palmitoyl ethanolamide	35047290	28213212	14173461	6044909	96968455	399523650
1212	Maltotetraose	3824928	324907052	619273	600085	3307762	3881598
1213	Gly-Phe	56817090	37094325	35293286	95990753	34884902	68891029

1214	FAHFA (18:2/20:4)	78678	74782028	73614	71306	11942090	14627222
1215	4-Hydroxy-L-Glutamic Acid	7033517	17499162	19914877	4948032	23441974	11312982
1216	2'-O-Methylguanosine	19141701	1117749	7923196	780248	1815390	62307963
1217	Dimethyl fumarate	900754430	385929486	355167111	427639911	466111884	571153151
1218	2-(Formylamino)Benzoic Acid	2729909	58168816	977570	10046049	66811557	80809545
1219	PC (11:0/12:0)	477111	330310286	1076036	590958	165623816	3812297
1221	ACar 18:0	471143	318400971	1008056	1069160	293607958	70188742
1222	ALK	2977294	291799095	4294506	7376242	3485599	3257307
1223	Orotidine	5427122	70112953	3917933	4706955	1098214	2277336
1224	Corchorifatty acid F	119080295	119502172	269467840	60252898	138245838	929559105
1225	2-Hydroxyvaleric acid	1114161960	79185263	2295367786	1818103433	1009041272	1003231191
1226	KKK	2156587	259872416	2477764	377110	3583875	2797854
1227	N-[3-(aminosulfonyl)phenyl]-2,3-dihydro-1,4-benzodioxine-2-carboxamide	1588793	68716379	706279	842997	3868996	2094970
1228	3-phenyl-5-[3-(trifluoromethyl)-1H-pyrazol-1-yl]-1,2,4-thiadiazole	2780928	17137883	12503225	45146890	310123621	142665765
1229	PC (16:2e/2:0)	21308016	25450915472	116184362	8459393	188067843	124847099
1230	5-[(Benzoyloxy)methyl]-4,5,6-trihydroxy-2-cyclohexen-1-yl benzoate	15665100	1200333	18635912	19466314	6893488	6978106
1231	LPE 14:0	1668725	313973050	25035762	1095552	106349497	9446592
1232	trans-Cinnamaldehyde	7457794	14328029	75623295	92140459	12713304	25639781
1233	LSD-d3	7075783	185428971	8340324	5824881	116907360	397572233
1234	Adenosine	19442334	2839537	4836468	3784724	3546857	3225464
1235	(2E)-N-(4-acetamidobutyl)-3-(4-hydroxy-3-methoxyphenyl)prop-2-enamide	19514810	5140991	12899410	11700703	6147648	6267232
1236	Indole	72979493	12268007	82251137	28853086	11941695	14539106

1237	Dodecanedioic acid	14771948	32734233	14560069	10889140	40187260	74834738
1238	Verbascose	623762	66858098	567240	470689	1946630	1436971
1239	methyl {[(2,4-difluoroanilino)carbothioyl]amino}methanimidate	176493	628544	1441769	10135064	59459354	59114048
1240	DKK	66803381	1134012	2772076	3054970	1572271	1258670
1241	PC (2:0/14:0)	269026	58390435	235410	329528	4837480	1427812
1242	Sorbic acid	73290375	18629937	61884259	58367284	30937397	43742678
1243	D-Phenylalanine	54894680352	27346750289	14674313788	58887315241	5032318749	5167120356
1244	N-[4-(diethylamino)phenyl]-N'-phenylurea	1639365678	412328397	105299386	814156367	17047112	2203526
1245	Fludrocortisone acetate	3249626	8047291	8258374	6699714	69487948	72495521
1246	Skatole	721438778	192656527	110205654	130811389	40555682	54911521
1247	7,8-dimethyl-1,2,3,4-tetrahydrophenazine	71874298	3474720	57241354	74378566	18776425	27549432
1248	1,2-Dipalmitoylphosphatidylglycerol	832355	305376955	885442	904617	261325829	9587110
1249	(5S)-5-hydroxy-1,7-diphenylheptan-3-one	60703289	1968076	33674496	30524036	4019468	5377864
1250	PC (18:4e/2:0)	4257057	3677434889	20218178	2967928	345523148	7405357
1251	QLH	70043076	6956877	45976003	59503619	3366533	5288329
1252	IQH	834954558	31149693	2403933	2670389	2778875	3463635
1253	PC (14:1e/5:0)	852545	302505909	1688782	1020567	119664876	28265724
1254	α-Aspartylphenylalanine	56022891	92294054	11876410	86753915	18043736	313870636
1255	Carvone	14364742	67731695	20270048	15536003	92684779	362655743
1256	Capryloylglycine	18278035	3514854	2686600	2084756	4011504	6728866
1257	trans-Cinnamic acid	2008362850	457429456	219491052	859499866	168989208	127909282
1258	LPC 19:1	119336	59224524	341265	125925	21328469	562146
1259	Bafilomycin B1	193220754	4362910	1029412596	181809638	210112023	16052832
1260	LPI 20:4	212225	924001397	345834	211836	2822047	1292165
1261	Ecgonine methyl ester	10749925	88848769	13995960	71849296	100925153	269189514

1262	LPC 8:0	3894214	285271039	4688123	6059262	71101982	61013729
1263	2-Methoxyestradiol	17728816	1777739	1856479	2499012	4942120	5653443
1264	R-1 Methanandamide phosphate	5657836	250720893	1579959	3228592	1350232	1409724
1265	1-(4-bromophenyl)-2-phenylethan-1-one	8344818	8210826	79893671	9033061	9336248	5660611
1266	Prostaglandin A1 ethyl ester	335165	4748916	297879	304865	25051228	58095710
1267	NMK	67529652	171266215	42520604	41696279	25177863	14214178
1268	4-methyl-5-oxo-2-pentyl-2,5-dihydrofuran-3-carboxylic acid	3625743	270578211	4376830	1561438	99796816	138535947
1269	6-Hydroxymelatonin	4472148	14502050	6253228	5065230	104311703	344224972
1270	lipoamide	16071318	4863796	15147612	17395023	14154668	10136613
1271	Proline-hydroxyproline	49901850	13251246	69202673	85092928	52061261	51653556
1272	6-methyl-7-nitro-2,3-dihydro-1,4-benzodioxine	8243359	12466563	11959013	9630909	32315471	355112488
1273	EPK	27502297	33631580	74386843	75732941	30527235	37005635
1274	13,14-dihydro-15-keto Prostaglandin D1	16719400	2954346	12500630	14933762	2255664	672379
1275	ACar 5:0	51679488	7460462	62491315	78903757	15758834	28657807
1276	PC (18:1e/4:0)	834191	281835456	1371764	1075218	163500033	45752474
1277	tetranor-PGFM	3651283	18453042	3437395	3305621	33965742	328765118
1278	2-(3,5-dimethyl-1H-pyrazol-4-yl)-5-methoxybenzoic acid	65841507	10786698	48494713	79792561	47693745	88874555
1279	Prostaglandin A1	251287075	8946440	44119451	80637977	20021317	5608695
1280	LPE 8:0	3996274	266101957	2193205	3493583	161226753	128779783
1281	Xanthosine	11191874	1225724	16462361	4759859	4322895	2459372
1282	11-Deoxy prostaglandin F1α	20828041	95850004	3478612	4874930	302596101	256813324
1283	PE (17:1/17:2)	955094	197244433	1151356	881223	180742656	15605860
1284	4-(pentyloxy)benzene-1-carbohydrazide	6347311	25569003	5919112	19791123	21526606	345434508
1285	N-Acetylneuraminic acid	3244689	264437882	4279048	1844258	6585729	11187234

1286	LPE 19:0	183957	61009353	216998	178336	9074467	1036531
1287	4-methylpyridine-3-sulfonic acid	14963230	15727479	10722762	11440502	82184790	314153963
1288	Vanillyl alcohol	1294170	10089889	1565889	1444811	9765642	65008539
1289	2-Thio-acetyl MAGE	33983404	5002817	68151588	673998	4744111	3548554
1290	Dactylorhin E	664470	1834023	405937	275590	1272787	59297502
1291	Thymine	178173650	553220098	932499238	1117380298	1309400111	26416202257
1292	3-Hydroxydecanoic acid	16948376	6356008	3576448	4348346	7189821	39989320
1293	NKK	1188664	233500781	1567031	3841233	1419330	1411899
1294	PC (2:0/16:0)	727110	788557041	882859	437844	18382791	1542625
